# Discovery of Natural Compound α‐Hederin via Large‐Scale Screening as a Targeted JAK/STAT3 Inhibitor for Ovarian Cancer Therapy

**DOI:** 10.1002/advs.202417278

**Published:** 2025-07-16

**Authors:** Jiayu Wang, Pengzhan He, Cheng Liu, Xin Chen, Yilin Tan, Rui Qu, Yan Zhang, Zhou Li, Tailang Yin, Zhinang Yin

**Affiliations:** ^1^ Department of Clinical Laboratory Institute of Translational Medicine Renmin Hospital of Wuhan University Wuhan Hubei 430060 China; ^2^ Department of Geriatrics Renmin Hospital of Wuhan University Wuhan Hubei 430060 China; ^3^ Department of Obstetrics and Gynecology Renmin Hospital of Wuhan University Wuhan Hubei 430060 China; ^4^ Reproductive Medical Center Renmin Hospital of Wuhan University Wuhan Hubei 430060 China; ^5^ School of Nanoscience and Engineering University of Chinese Academy of Sciences Beijing 101400 China

**Keywords:** α‐Hederin, JAK, proliferation, metastasis, ovarian cancer

## Abstract

Chemoresistance and metastasis are key obstacles to successful ovarian cancer (OC) treatment. Here, α‐Hederin, a pentacyclic triterpenoid saponin, is identified as a potent and selective dual inhibitor of JAK1/JAK2 with promising therapeutic potential in OC. Integrating transcriptomic analysis, virtual screening, molecular docking, and biochemical validation, it is shown that α‐Hederin directly binds the JH1 kinase domains of JAK1 and JAK2, suppressing their activity and downstream STAT3 phosphorylation. α‐Hederin inhibits OC cell proliferation, epithelial‐mesenchymal transition (EMT), and metastasis in vitro, and suppresses tumor growth and dissemination in multiple mouse models, with minimal systemic toxicity. Mechanistically, α‐Hederin blocks STAT3 nuclear translocation and downregulates oncogenic STAT3 targets including MYC, CCND1, and TWIST1. Rescue experiments using the STAT3 agonist Colivelin partially reversed these effects, confirming the JAK/STAT3 axis as a key target. Moreover, α‐Hederin synergizes with cisplatin to enhance antitumor efficacy and overcomes platinum resistance in OC cells. Collectively, our findings highlight α‐Hederin as a safe and effective natural JAK1/2 inhibitor that suppresses OC progression by targeting the JAK/STAT3 pathway, offering a compelling candidate for future clinical translation.

## Graphical abstract

This study identifies α‐Hederin as a potent dual JAK1/JAK2 inhibitor that blocks STAT3 activation in ovarian cancer. By disrupting STAT3‐driven transcriptional programs, α‐Hederin suppresses tumor proliferation, invasion, and EMT, while enhancing cisplatin efficacy and overcoming chemoresistance. These findings highlight a promising natural compound‐based strategy for targeting JAK/STAT3 signaling in ovarian cancer therapy.

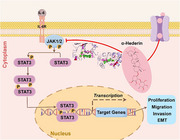

## 1 Introduction

Ovarian cancer (OC) is a prevalent and highly aggressive malignancy of the female reproductive system. Ranking third in incidence among gynecological cancers but first in mortality, OC poses a severe health threat.^[^ ^1^ ^]^ Standard treatments for OC include tumor debulking surgery and postoperative platinum‐based chemotherapy,^[^ ^2^ ^]^ which often achieve initial remission but are hampered by high rates of drug resistance and tumor recurrence.^[^ ^3^ ^]^ The 5‐year survival rate for OC is less than 40%.^[^ ^4^, ^5^ ^]^ The toxicity of current therapies to normal tissues and the challenge of overcoming chemotherapy resistance emphasize the need for novel therapeutic approaches.^[^ ^6^ ^]^

Recent molecular studies have identified the JAK/STAT3 signaling pathway as a pivotal driver of OC progression and treatment resistance. Constitutive activation of JAK1/JAK2‐mediated STAT3 phosphorylation facilitates tumor cell proliferation, survival, metastasis, immune evasion, and chemoresistance.^[^ ^7^, ^8^, ^9^, ^10^ ^]^ While small‐molecule JAK inhibitors (e.g., Ruxolitinib and Fedratinib) have been developed, their clinical applications are limited by adverse effects such as myelosuppression and immunosuppression.^[^ ^11^ ^]^ Therefore, identifying safer, naturally derived JAK/STAT3 pathway inhibitors represents a promising therapeutic strategy in OC management.

Natural products are increasingly being explored for their anticancer potential due to their structural diversity, bioavailability, and low toxicity.^[^ ^12^ ^]^ Among them, α‐Hederin, a pentacyclic triterpenoid saponin isolated from Hedera helix L. and Nigella sativa, has shown antitumor activities in several cancer types, including hepatocellular carcinoma and colon cancer.^[^ ^13^, ^14^ ^]^ Mechanistically, α‐Hederin has been reported to modulate oxidative stress pathways, block tumor‐related signaling cascades, and induce apoptosis via ROS‐mediated mitochondrial dysfunction.^[^ ^15^, ^16^, ^17^, ^18^, ^19^ ^]^ However, its potential role and underlying mechanisms in OC, particularly in targeting the JAK/STAT3 axis, have not yet been comprehensively elucidated.

In this study, we aim to systematically investigate the therapeutic efficacy of α‐Hederin in OC, with a particular focus on its effects on the JAK1/JAK2‐STAT3 signaling pathway. By integrating computational drug screening, molecular docking, and extensive in vitro and in vivo validations, we provide strong preclinical evidence that α‐Hederin selectively inhibits the JAK/STAT3 axis, thereby suppressing OC cell proliferation, invasion, metastasis, and epithelial‐mesenchymal transition (EMT). This work not only highlights a novel application of α‐Hederin as a dual JAK1/JAK2 inhibitor, but also contributes to the development of low‐toxicity, natural compound‐based strategies for overcoming resistance and improving OC treatment outcomes.

## 2 Results

### 2.1 Multiscale Drug Screening Identifies Anti‐OC Compounds

**Figure 1:**
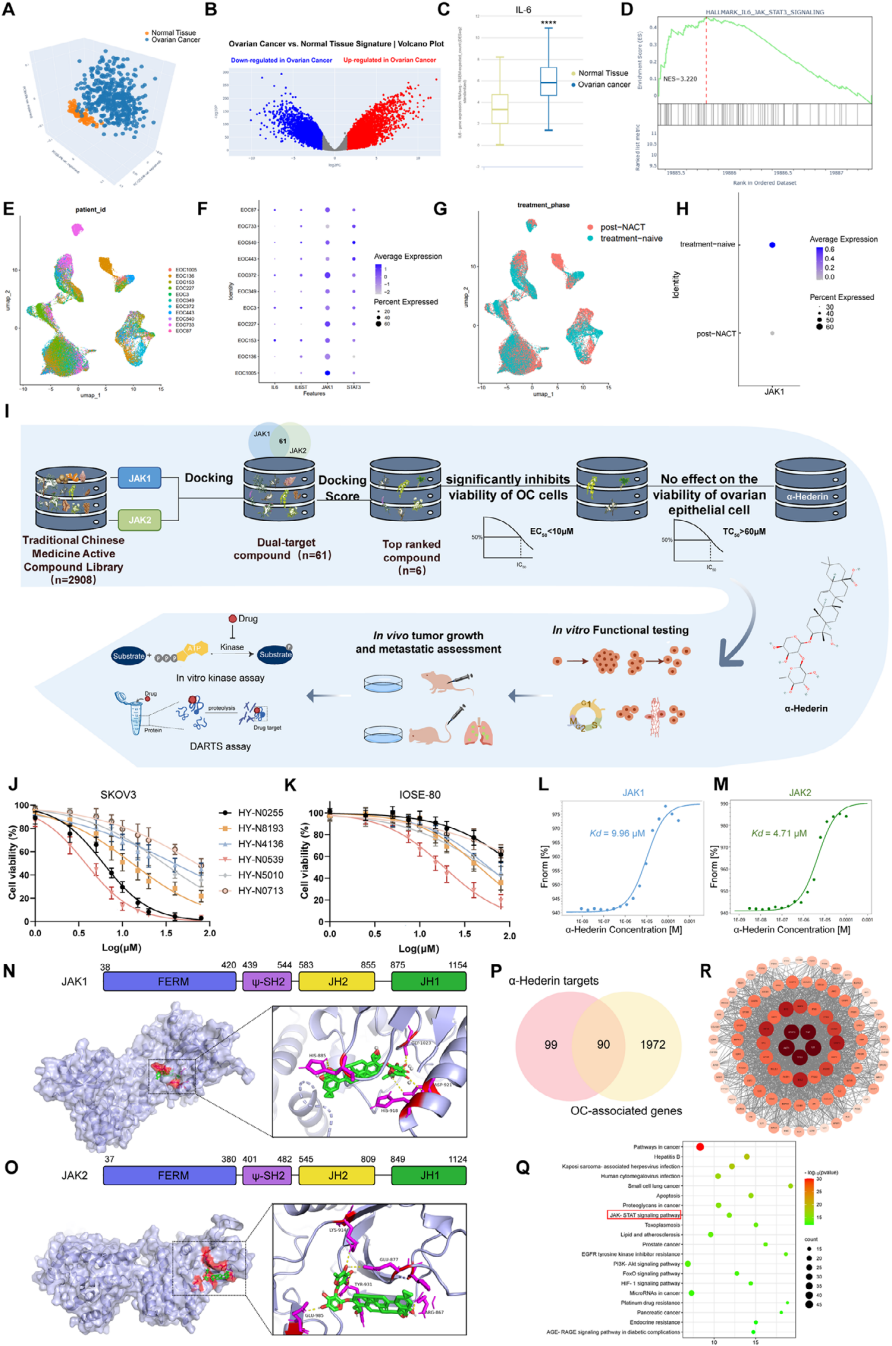
Multiscale analysis identifies α‐Hederin as a JAK/STAT3‐targeting compound for OC. (A) 3D Principal component analysis (PCA). PCA plot was generated using the 2500 genes with the highest variance across samples. Normal tissues are represented by orange points, while OC samples are indicated by blue points. (B) Volcano plot showing differentially expressed genes between OC and normal tissues. (C) Boxplot showing increased mRNA expression of IL‐6 in OC tissues compared to normal tissues, based on TCGA and GTEx datasets. (D) GSEA indicating significant enrichment of the IL‐6/JAK/STAT3 signaling pathway in OC. (E) Uniform Manifold Approximation and Projection (UMAP) plot of 51 786 single cells from 11 epithelial ovarian cancer (EOC) patients (GSE165897), color‐coded by patient identity. (F) Expression levels of IL6, IL6ST, JAK1, and STAT3 across single‐cell populations. (G) UMAP plots show the distribution of cells before and after NACT treatment. (H) JAK1 expression levels before and after NACT treatment. (I) Workflow of structure‐based virtual screening of 2908 natural compounds targeting JAK1 and JAK2, followed by cytotoxicity validation in OC and normal ovarian epithelial cells. (J) Dose‐response curves quantifying viability of OC cells upon drug treatment for 48 h. The code names of drugs are listed on the right. (K) Dose‐response curves quantifying viability of ovarian epithelial cells upon drug treatment for 48 h. (L) Binding affinity measurements of α‐Hederin and JAK1 as measured via MST thermophoresis curve analysis. (M) Binding affinity measurements of α‐Hederin and JAK2 as measured via MST thermophoresis curve analysis. (N) Schematic structures of JAK1. Molecular docking results of α‐Hederin (green) with JAK1 (blue). The docking sites of α‐Hederin on JAK1 were highlighted in magenta. (O) Schematic structures of JAK2. Molecular docking results of α‐Hederin (green) with JAK2 (blue). The docking sites of α‐Hederin on JAK2 were highlighted in magenta. (P) Venn diagram displaying α‐Hederin targets (pink) and OC‐associated genes (yellow). The overlapping regions indicate common targets. (Q) KEGG analysis highlighted the top 20 pathways with significant enrichment. Then red box indicated the JAK/STAT3 signaling pathway. (R)The schematic diagram of the drug‐target gene network was visualized using Cytoscape software.

By analyzing the Genotype‐Tissue Expression database (GTEx) and The Cancer Genome Atlas (TCGA) datasets via the UCSC Xena Browser,^[^ ^20^ ^]^ we conducted a comparative analysis of mRNA expression profiles between 419 primary OC tissues and 88 normal ovarian tissues (**Figure** **1**A,B). Our results revealed a marked upregulation of IL‐6 (Figure 1C) and IL‐6R (Figure S1A, Supporting Information) in OC tissues compared to normal tissues, suggesting an active role of these markers in OC pathogenesis. Furthermore, GSEA identified excessive activation of the IL6/JAK/STAT3 signaling pathway in tumor tissues (Figure 1D; Figure S1B, Supporting Information), corroborating previous findings in the literature.^[^ ^21^, ^22^ ^]^ We analyzed single‐cell sequencing data from 11 OC patients in the GSE165897 dataset (Figure 1E) and observed high expression levels of IL6, IL6ST, JAK1, and STAT3 in OC (Figure 1F; Figure S1C, Supporting Information).^[^ ^23^ ^]^ Cells were annotated based on pre‐and post‐neoadjuvant chemotherapy (NACT) treatment (Figure 1G), revealing a significant downregulation of JAK1 expression following treatment (Figure 1H). These results underscored the potential of targeting JAK activity as an effective therapeutic strategy for OC.

Given the immense potential of natural compounds in cancer treatment, we implemented a structure‐based computational screening approach followed by cytotoxicity assays (Figure 1I). We conducted a computational screening of over 2900 natural compounds targeting JAK1 and JAK2 proteins, selecting the top candidates based on docking scores. To refine the selection, we ranked the compounds separately for JAK1 and JAK2, identifying the top 200 compounds for each protein. From these, we selected the intersecting compounds that appeared in both rankings and ensured that their docking scores were less than ‐12.0 for both JAK1 and JAK2. These six top‐performing compounds were further assessed for their cytotoxicity in OC cells (SKOV3) across nine concentration gradients ranging from 0 to 80 µm over a 48‐h incubation period. Effective concentration (EC_50_) values were determined in SKOV3 cells, while toxicity concentration (TC_50_) values were evaluated in human ovarian epithelial cells (IOSE‐80). Among the screened compounds, α‐Hederin (HY‐N0255) was identified as the most promising candidate, exhibiting an EC50 of 6.05 µm (Figure 1J) with no detectable toxicity to ovarian epithelial cells at concentrations up to 60 µm (Figure 1K).

Robust binding curves were observed for α‐Hederin with JAK1 and JAK2, with Kd values of 9.96 µm for JAK1 (Figure 1L) and 4.71 µm for JAK2 (Figure 1M). Binding curves for the remaining five compounds with JAK1 and JAK2 were observed (Figure S1D, Supporting Information). Figure 1N depicts the structures of JAK1. Docking analysis with AutoDock Vina confirmed the strong binding affinity of α‐Hederin to the JH1 kinase domain of JAK1, with a binding free energy of −12.57 kcal mol^−1^. Similarly, α‐Hederin was found to bind to the JH1 kinase domain of JAK2, with a binding free energy of −12.10 kcal mol^−1^ (Figure 1O), further supporting its potential as a dual‐target inhibitor. PyMOL visualization showed that α‐Hederin formed hydrogen bonds with HIS‐885, HIS‐918, ASP‐921, and GLY‐1023 of JAK1 (Figure 1N and Table S1, Supporting Information), while it formed hydrogen bonds with ARG‐867, GLU‐877, LYS‐914, TYR‐931, and GLU‐985 of JAK2 (Figure 1O and Table S2, Supporting Information).

To further verify the potential targets of α‐Hederin in OC, we conducted a network pharmacology analysis. A total of 189 α‐Hederin targets were identified from PubChem Bioassay and 2062 OC targets from databases including GeneCards, DisGeNET, and OMIM. The analysis identified 90 overlapping targets between α‐Hederin and OC (Figure 1P). KEGG analysis revealed enrichment in cancer‐associated pathways, including JAK/STAT, PI3K‐Akt, and HIF‐1 signaling (Figure 1Q). Cytoscape identified STAT3 as a key factor (Figure 1R). The overactivation of the JAK/STAT3 pathway in OC (Figure S1E, Supporting Information) supports its critical role in α‐Hederin's anti‐OC effects.

### 2.2 α‐Hederin Suppresses OC Proliferation In Vitro

**Figure 2:**
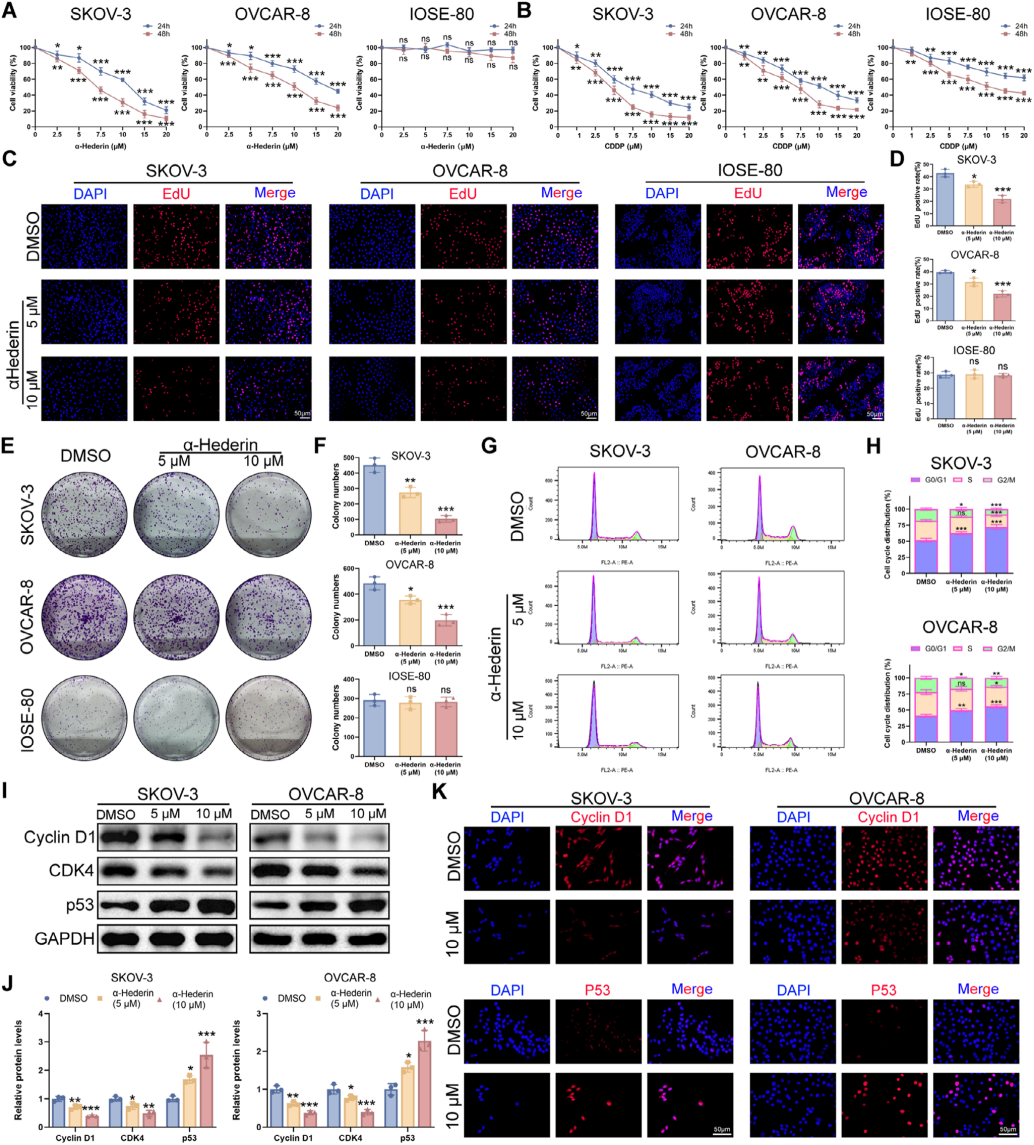
α‐Hederin inhibits the proliferation of OC cells without affecting IOSE‐80 cells. (A,B) Cell viability of SKOV‐3, OVCAR‐8, and IOSE‐80 cells treated with increasing concentrations of α‐Hederin (A) or CDDP (B) for 24 and 48 h, measured by the CCK‐8 assay. (C) Representative images of EdU staining in cells treated with 0, 5, or 10 µm α‐Hederin for 48 h to assess proliferation. Scale bar: 50 µm. (D) Quantification of EdU‐positive cells. (E) Representative images of colony formation in SKOV‐3, OVCAR‐8, and IOSE‐80 cells treated with α‐Hederin. (F) Quantification of colony numbers from (E). (G) Representative flow cytometry plots of the cell cycle distribution in SKOV‐3 and OVCAR‐8 cells following treatment with 0, 5, or 10 µm α‐Hederin for 48h. (H) Quantification of cell cycle phase proportions in (G). (I) Western blot analysis of Cyclin D1, CDK4, and p53 expression in response to α‐Hederin at 5 or 10 µm for 48 h. (J) Quantification of protein levels from (I). (K) IF staining of Cyclin D1 and p53 in cells treated with 0 or 10 µm α‐Hederin. Scale bar: 50 µm. Data are presented as mean ± SD from at least three independent experiments. Statistical significance was evaluated using unpaired two‐tailed Student's *t*‐test for two‐group comparisons and one‐way ANOVA for multiple group comparisons. Compared to DMSO: *p < 0.05, **p < 0.01, ***p < 0.001, ns: not significant.

The CCK‐8 assay was first conducted to evaluate the impact of α‐Hederin on OC cells (SKOV‐3 and OVCAR‐8 cells) and normal ovarian cells (IOSE‐80). Cisplatin (CDDP) was added as a positive control. α‐Hederin significantly inhibited OC cell proliferation in a time‐ and dose‐dependent manner. Although α‐Hederin required a higher dosage than CDDP to achieve similar effects, we found that α‐Hederin exhibits almost no toxicity to normal ovarian cells (**Figure** **2**A,B). 5 µm α‐Hederin showed significant anti‐tumor activity against OC cells after 48 h of treatment. Thus, SKOV‐3 and OVCAR‐8 cells were treated with 5 and 10 µm of α‐Hederin for 48 h to further explore the proliferation ability. EdU experiments demonstrated a significant decrease in SKOV‐3 and OVCAR‐8 cell proliferation rate when treated with increasing α‐Hederin concentrations (Figure 2C,D) or CDDP (Figure S2A,B, Supporting Information). Colony formation assays revealed a dose‐dependent reduction of colony numbers (Figure 2E,F). This effect is also consistent with the CDDP, positive control (Figure S2C,D, Supporting Information). Cell proliferation often correlates with alterations in cell cycle progression.^[^ ^24^ ^]^ Flow cytometry showed that α‐Hederin arrested OC cells in the G0/G1 phase (Figure 2G,H). This was accompanied by a significant reduction in the levels of Cyclin D1 and CDK4, the key effectors in the regulation of cell cycle G1/S transition. α‐Hederin also increased the levels of p53, a tumor suppressor halting the cell cycle in the G1 phase (Figure 2I–K). Similar results were observed with CDDP (Figure S2E–J, Supporting Information). These findings suggest that α‐Hederin impedes the cell cycle in OC cells by targeting crucial regulatory proteins.

### 2.3 α‐Hederin Inhibits OC Cell Migration and Invasion

**Figure 3:**
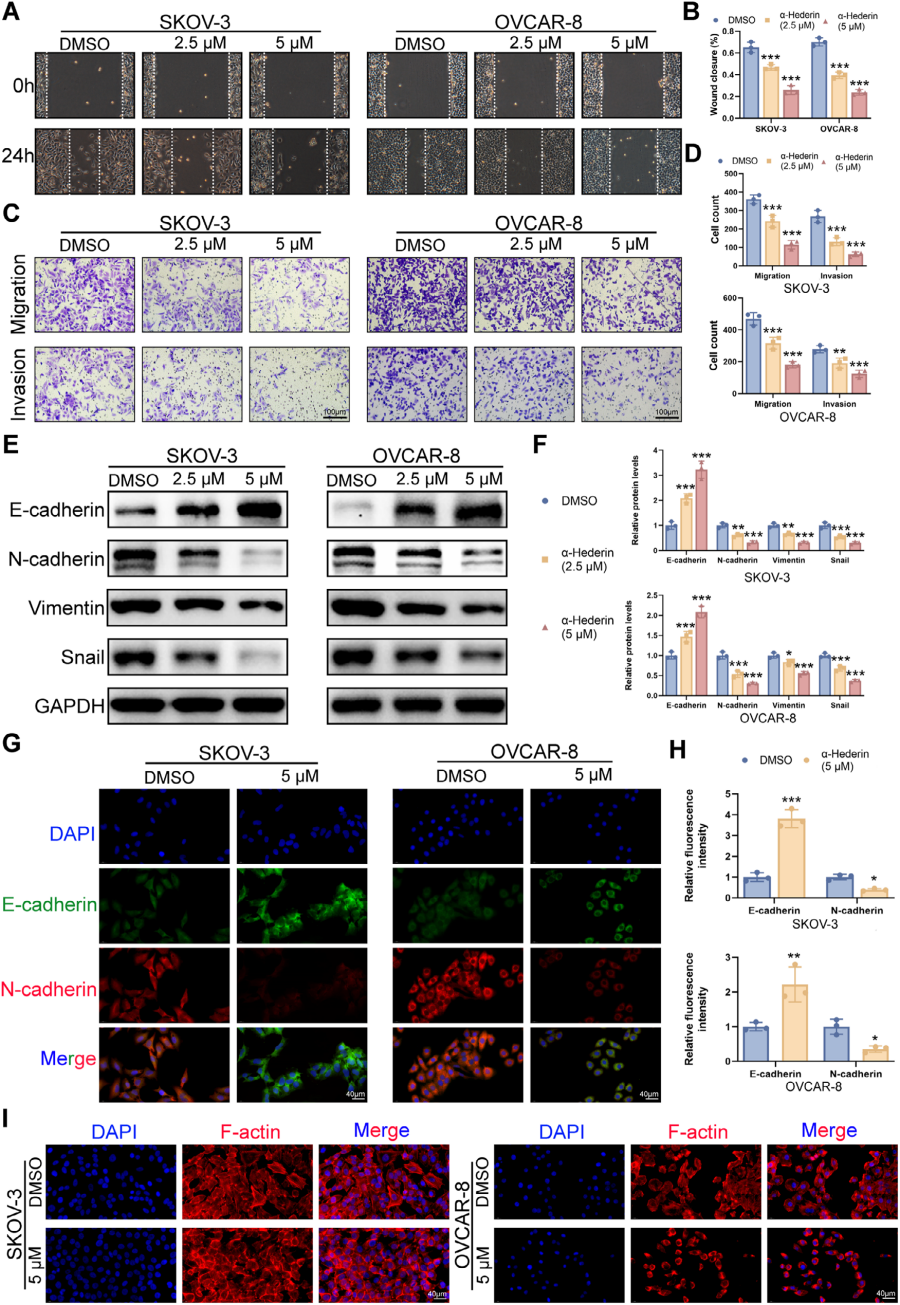
α‐Hederin suppresses the migration and invasion of OC cells in vitro. (A) Representative images from wound healing assays showing the migration of SKOV‐3 and OVCAR‐8 cells treated with α‐Hederin (2.5 or 5 µm) for 24 h. White lines indicate the wound edges. (B) Quantification of wound closure percentage relative to the initial gap width. (C) Representative Transwell images showing the effects of α‐Hederin on cell migration (upper panels) and invasion (lower panels) in SKOV‐3 and OVCAR‐8 cells. (D) Quantification of migrated and invaded cells in (C). (E) Western blot analysis of EMT‐related markers (E‐cadherin, N‐cadherin, Vimentin, and Snail) in cells treated with increasing concentrations of α‐Hederin. (F) Quantification of protein levels from (E). (G) Representative IF images of E‐cadherin and N‐cadherin in SKOV‐3 and OVCAR‐8 cells treated with 5 µm α‐Hederin for 24 h. Scale bar: 40 µm. (H) Quantification of fluorescence intensity from (G). (I) Confocal fluorescence images of F‐actin (red) and nuclei (DAPI). Scale bar: 40 µm. Data are presented as mean ± SD of at least three independent experiments. Statistical significance was evaluated using unpaired two‐tailed *t*‐tests for pairwise comparisons and one‐way ANOVA for multiple group comparisons. Compared to DMSO: *p < 0.05, **p < 0.01, ***p < 0.001.

Migration and invasion are important mechanisms aside from proliferation for tumor development. To evaluate the effects of α‐Hederin (2.5 and 5 µm for 24 h) on OC cell migration and invasion, we conducted Cell Scratch and Transwell assays. Treatment with α‐Hederin at these concentrations for 24 h maintained OC cell viability at ≈90%, ensuring that the observed results were not influenced by cell growth. The results revealed a significant and dose‐dependent inhibition of migration and invasion (**Figure** **3**A–D) in SKOV‐3 and OVCAR‐8 cells treated with α‐Hederin compared to the controls. Epithelial‐mesenchymal transition (EMT) is a key feature of metastasis, marked by enhanced cell migration and invasion. Treatment with α‐Hederin led to an increase in the epithelial marker E‐cadherin and a decrease in mesenchymal markers such as Vimentin, N‐cadherin, and the EMT‐associated transcription factor Snail (Figure 3E,F). IF staining further confirmed these results, showing elevated E‐cadherin and reduced N‐cadherin in OC cells treated with α‐Hederin (Figure 3G,H). Additionally, CDDP, used as a positive control, significantly reduced cell migration, invasion, and the EMT process in SKOV‐3 and OVCAR‐8 cells (Figure S3A–H, Supporting Information). F‐actin staining revealed that α‐Hederin treatment suppressed the progression of EMT, as evidenced by the reduced formation of stress fibers and filopodia, which are hallmarks of mesenchymal cell morphology (Figure 3I). These observations underscored the role of α‐Hederin in suppressing migration and invasion in OC cells and provide insights into its underlying mechanisms.

### 2.4 α‐Hederin Reduces OC Cell Growth and Metastasis In Vivo

**Figure 4:**
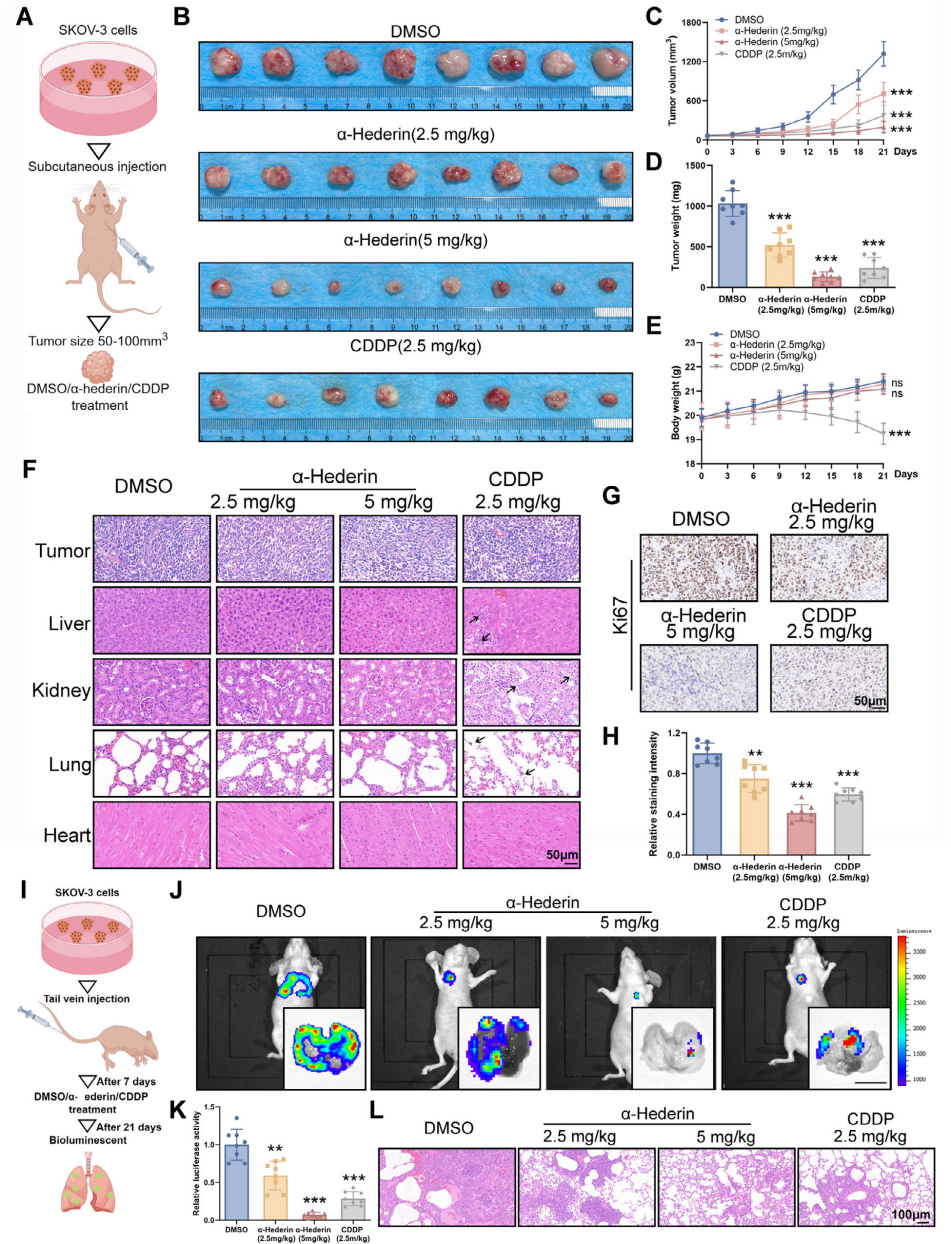
α‐Hederin inhibits tumor growth and metastasis of OC in vivo. (A) Schematic illustration of the subcutaneous xenograft model. (B) Representative images of tumors from mice treated with DMSO, α‐Hederin (2.5 or 5 mg kg^−1^), or CDDP (2.5 mg kg^−1^). (C) Tumor volume was measured every 3 days. (D) Tumor weight at the endpoint of the experiment. (E) Body weight of mice was monitored throughout the study. (F) H&E staining of tumors, liver, kidney, lung, and heart tissues to evaluate histopathological changes. Scale bar: 50 µm. (G) Representative IHC images of Ki67 staining in tumor sections. Scale bar: 50 µm. (H) Quantitative analysis of Ki67 staining intensity. (I) Schematic illustration of the lung metastasis model. (J) Representative bioluminescence imaging of whole mice and isolated lungs at day 21, showing metastatic burden. (K) Quantification of lung luciferase activity. (L) H&E staining of lung tissues showing metastatic nodules. Scale bar: 100 µm. Data are presented as mean ± SD from eight mice per group (n = 8). Statistical significance was assessed using one‐way ANOVA for multiple group comparisons. Compared to DMSO: **p < 0.01, ***p < 0.001, ns: not significant.

To assess the impact of α‐Hederin on tumor growth in vivo, OC xenografts were subcutaneously implanted into BALB/c nude mice and permitted to grow to a volume of ≈50–100 mm^3^. Tumor‐bearing mice were treated with α‐Hederin or CDDP (**Figure** **4**A). Tumor growth was notably inhibited in both drugs (Figure 4B). Both the α‐Hederin group and CDDP group exhibited a significantly smaller tumor volume and markedly lower tumor weight compared to the control group (Figure 4C,D). Meanwhile, there were no significant differences between DMSO (control group) and α‐Hederin‐treated mice in terms of body weight, in contrast with the significant body loss caused by CDDP (Figure 4E). Histological examination of vital organs, including the liver, kidney, lung, and heart, did not reveal any overt morphological changes in α‐Hederin‐treated group (Figure 4F). CDDP group showed observable vacuolar and inflammatory cell infiltration in the liver and kidneys, and widened alveolar septa in the lungs (Figure 4F). The results of liver and kidney function tests revealed notable abnormalities in the CDDP group, whereas the α‐Hederin and DMSO groups did not exhibit any discernible alterations. Unlike CDDP, which induced notable hematopoietic suppression as evidenced by decreased RBC, WBC, HGB, and PLT counts, α‐Hederin maintained these parameters at physiological levels, indicating minimal impact on bone marrow hematopoiesis (Figure S4A, Supporting Information). As the Ki67 percentage was reduced significantly in tumors treated with α‐Hederin, the reduced tumor mass is likely related to inhibition of the proliferation of tumor cells. (Figure 4G,H). Western blot and IHC analyses of the xenograft model further confirmed these findings. Cell cycle‐related proteins and EMT markers exhibited comparable alterations to those observed in the in vitro model (Figure S4B–E, Supporting Information).

Further, a lung metastasis model was evaluated using bioluminescence imaging to reveal the role of α‐Hederin in OC cell metastasis (Figure 4I). Bioluminescence imaging indicated that α‐Hederin inhibited lung metastasis of OC cells (Figure 4J,K). These results were confirmed by H&E staining of the lungs (Figure 4L).

### 2.5 α‐Hederin Inhibits the STAT3 Pathway by Targeting JAK1 and JAK2

**Figure 5:**
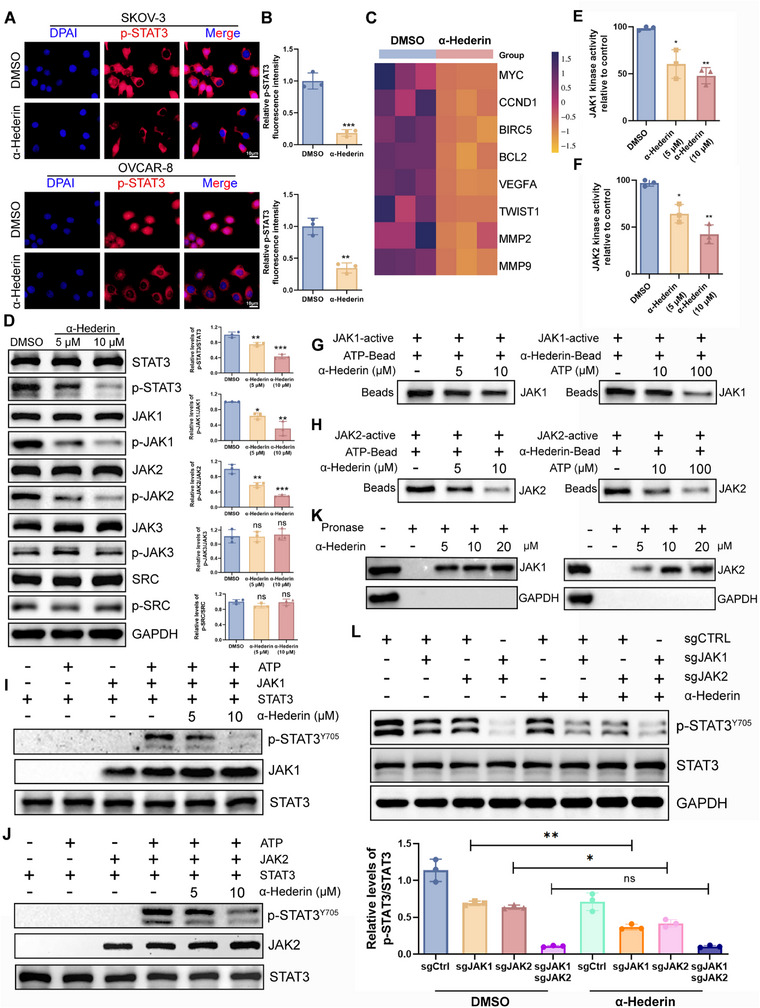
α‐Hederin directly binds to JAK1/2 and inhibits STAT3 phosphorylation and nuclear translocation. (A,B) IF staining of p‐STAT3 and statistical analysis of fluorescence intensity. Scale bar: 10 µm. (C) Heatmap showing relative mRNA expression of STAT3 downstream targets (MYC, CCND1, BIRC5, BCL2, VEGFA, TWIST1, MMP2, and MMP9) following α‐Hederin treatment, measured by qRT‐PCR and normalized to GAPDH. (D) Western blot analysis of total and phosphorylated STAT3, JAK1, JAK2, JAK3, and SRC in SKOV‐3 cells treated with α‐Hederin (5 or 10 µm) or DMSO. Statistical analysis is presented. (E,F) Kinase assay to examine the effect of α‐Hederin on JAK1 (E) and JAK2 (F) kinase activity. (G,H) The competitive binding relationship between α‐Hederin and ATP was confirmed using a pull‐down assay. (I,J) In vitro kinase assays were performed using bacterial‐purified His‐STAT3 and the active JAK1 (I) and JAK2 (J) kinase. The amount of α‐Hederin in the reaction is indicated. (K) DARTS (drug affinity responsive target stability) assay showing α‐Hederin‐mediated stabilization of JAK1 and JAK2 proteins in SKOV‐3 lysates. (L) Western blot analysis of p‐STAT3 and total STAT3 in SKOV‐3 cells with sgCtrl, sgJAK1, sgJAK2, or sgJAK1+sgJAK2, treated or not with 5 µm α‐Hederin. Bottom panel: quantification of p‐STAT3/STAT3 ratio. Data are presented as mean ± SD from at least three independent experiments. Statistical significance was determined by unpaired two‐tailed Student's *t*‐test for two‐group comparisons and one‐way ANOVA for comparisons among multiple groups. *p < 0.05, **p < 0.01, ***p < 0.001, ns: not significant.

As demonstrated earlier, α‐Hederin bonded to the JH1 domain of both JAK1 and JAK2, a region essential for controlling kinase activity. Building on this, we investigated whether α‐Hederin affects STAT3, a major substrate of JAK kinases. **Figure** **5**A,B showed that α‐Hederin reduced the phosphorylation of STAT3 and nuclear accumulation as shown by IF. As a result of α‐Hederin treatment, STAT3‐targeting genes associated with malignant biological behaviors were decreased in OC cells. These include MYC, CCND1, BIRC5, BCL2, VEGFA, TWIST1, MMP2, and MMP9 (Figure 5C). The expression and phosphorylation levels of STAT3 at Y705 and upstream kinase were investigated after α‐Hederin treatment to further reveal its effects on p‐STAT3 inhibition. A significant reduction in p‐STAT3^Y705^ was observed in OC cells without alteration in STAT3 protein expression following α‐Hederin treatment. The upstream kinase affected by α‐Hederin was JAK1 and JAK2, but not JAK3 or SRC (Figure 5D). Animal experiments have also confirmed that the administration of α‐Hederin can significantly inhibit the phosphorylation levels of STAT3, JAK1, and JAK2 in tumors in a dose‐dependent manner (Figure S5A–D, Supporting Information). α‐Hederin reduced the kinase activity of JAK1 (Figure 5E) and JAK2 (Figure 5F) in a concentration‐dependent manner. A pull‐down assay with ATP or α‐Hederin agarose beads was performed to assess the effect of α‐Hederin on the ability of JAK1 and JAK2 to bind to ATP. Results showed α‐Hederin significantly decreased JAK1 and JAK2 binding to ATP dose‐dependently (Figure 5G,H). Additionally, an in vitro kinase assay was conducted using recombinant JAK1 and JAK2, both with and without α‐Hederin. α‐Hederin diminished the ability of JAK1 and JAK2 to phosphorylate STAT3 (Figure 5I,J). DARTS is a method that leverages the decreased protease sensitivity of target proteins after they bind to a drug. Once the proteins interact with the drug, they become resistant to degradation by pronase. SKOV‐3 cell lysates were first incubated with α‐Hederin and then digested with pronase. Proteins that bind to α‐Hederin can be protected from pronase degradation. α‐Hederin protected both JAK1 and JAK2 from degradation in a dose‐dependent manner (Figure 5K). We then performed targeted gene knockdown of JAK1 and JAK2 in SKOV3 OC cells using CRISPR/Cas9 (Figure S6A,B, Supporting Information). As shown in Figure 5L, 5 µm α‐Hederin treatment significantly suppressed STAT3 phosphorylation in sgCtrl cells. Knockdown of either JAK1 or JAK2 alone partially reduced p‐STAT3 levels, but did not abolish α‐Hederin's inhibitory effect. Notably, dual knockdown of JAK1 and JAK2 led to a marked reduction in baseline p‐STAT3 expression, and in this context, α‐Hederin treatment failed to further suppress STAT3 phosphorylation. To further investigate the specificity of α‐Hederin in modulating oncogenic signaling pathways in OC, we examined its effect on other canonical pathways including PI3K/AKT, MAPK, and NF‐κB. α‐Hederin treatment did not significantly alter the phosphorylation levels of AKT, ERK1/2, or NF‐κB p65 compared to control, indicating that α‐Hederin neither activates nor inhibits these signaling cascades (Figure S6C,D, Supporting Information). To exclude the potential involvement of alternative pathways, SKOV‐3 and OVCAR‐8 cells were pretreated with selective inhibitors, including the JAK1/2 inhibitor Ruxolitinib, the STAT3 inhibitor Stattic, the PI3K inhibitor LY294002, and the HIF‐1α inhibitor PX‐478, followed by α‐Hederin exposure. As shown in Figure S6E–H (Supporting Information), pretreatment with LY294002 and PX‐478 markedly enhanced the inhibitory effects of α‐Hederin on cell proliferation, migration, and invasion, suggesting additive or synergistic interactions. In contrast, neither Ruxolitinib nor Stattic enhanced α‐Hederin's effects. These findings indicate that α‐Hederin inhibits STAT3 activation predominantly through targeting both JAK1 and JAK2.

### 2.6 α‐Hederin's Anti‐Cancer Effects were Partially Reversed by STAT3 Reactivation In Vivo and In Vitro

**Figure 6:**
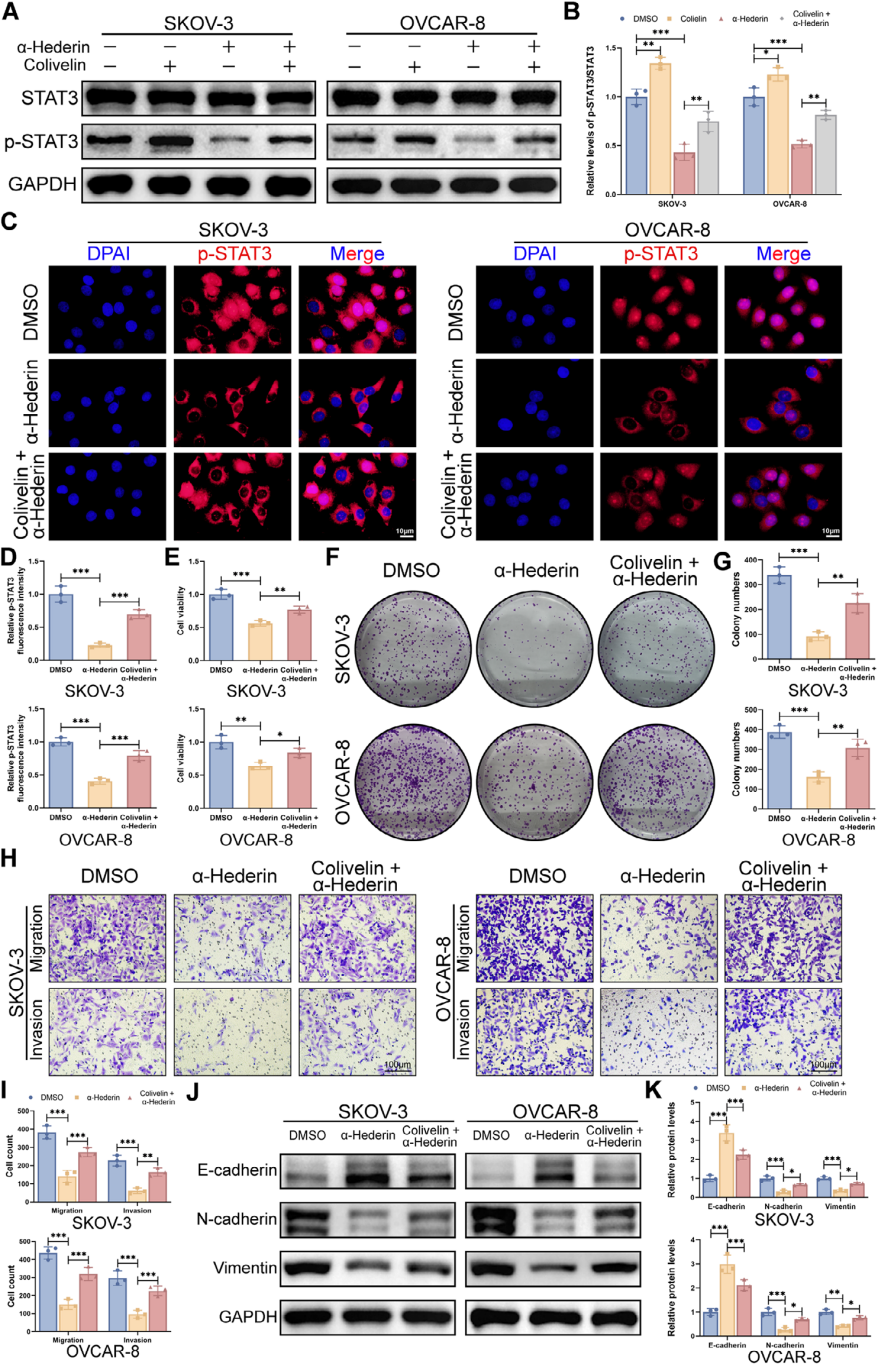
STAT3 reactivation partially reverses the anti‐tumor effects of α‐Hederin in OC cells in vitro. (A) Western blot analysis of total and phosphorylated STAT3 in SKOV‐3 and OVCAR‐8 cells treated with α‐Hederin (10 µm), Colivelin (0.5 µm), or both for 24 h. (B) Quantification of p‐STAT3/STAT3 levels from (A). (C) IF staining of p‐STAT3 in SKOV‐3 and OVCAR‐8 cells treated as in (A). Scale bar: 10 µm. (D) Quantification of fluorescence intensity from (C). (E) Cell viability was measured by CCK‐8 assay in the same treatment groups. (F) The colony formation assay was used to detect the proliferation of SKOV‐3 and OVCAR‐8 cells treated with α‐Hederin for 48h with or without Colivelin. (G) Quantification of colony numbers from (F). (H) Representative Transwell images showing cell migration (top) and invasion (bottom) in SKOV‐3 and OVCAR‐8 cells under the indicated treatments. Scale bar: 100 µm. (I) Quantitative analysis of migrated and invaded cells from (H). (J) Western blot analysis of EMT markers (E‐cadherin, N‐cadherin, and Vimentin) in cells treated with α‐Hederin and/or Colivelin. (K) quantification of protein levels from (J). Data are presented as mean ± SD from three independent experiments. Statistical significance was determined using one‐way ANOVA for multiple group comparisons. *p < 0.05, **p < 0.01, ***p < 0.001.

To better identify whether α‐Hederin inhibited OC via the JAK/STAT3 pathway, we conducted rescue experiments with Colivelin, an activator of STAT3.^[^ ^25^ ^]^ Colivelin markedly elevated p‐STAT3^Y705^ levels in both α‐Hederin‐treated and untreated OC cells, as demonstrated by Western blot (**Figure** **6**A,B) and IF analysis (Figure 6C,D). We then assessed whether reactivating STAT3 would influence the phenotypic changes induced by α‐Hederin in OC cells. CCK‐8 and colony formation assays revealed that Colivelin partially countered the proliferation‐inhibiting effects of α‐Hederin (Figure 6E–G). Furthermore, Colivelin enhanced the migration and invasion of α‐Hederin‐treated SKOV‐3 and OVCAR‐8 cells (Figure 6H,I). Colivelin also significantly reversed the EMT changes caused by α‐Hederin (Figure 6J,K). These demonstrated that STAT3 activator significantly restored the migration and invasion ability of α‐Hederin‐treated SKOV‐3 and OVCAR‐8 cells.

**Figure 7:**
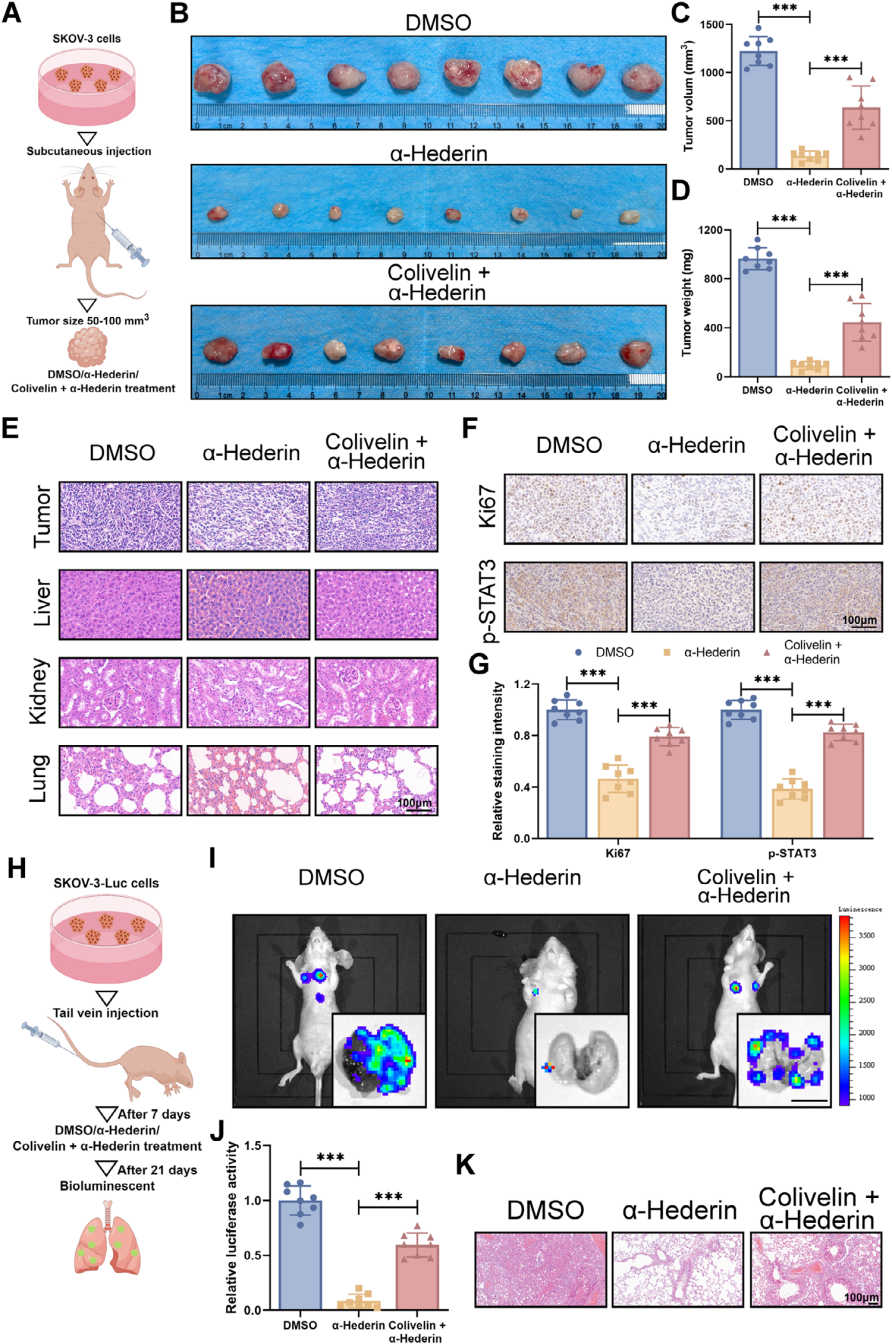
STAT3 reactivation partially reverses the anti‐tumor effects of α‐Hederin in vivo. (A) Schematic illustration of the subcutaneous xenograft model. (B) Representative images of tumors from mice treated with DMSO, α‐Hederin, or α‐Hederin + Colivelin. (C) Tumor volume was measured every 3 days. (D) Tumor weight at the endpoint of the experiment. (E) H&E staining of tumor, liver, kidney, and lung tissues to evaluate histopathological changes. Scale bar: 100 µm. (F,G) IHC staining of p‐STAT3 and Ki67, along with quantitative analysis of staining intensity. Scale bar: 100 µm. (H) Schematic illustration of the lung metastasis model. (I) Representative bioluminescence imaging of whole mice and isolated lungs showing metastatic burden. (J) Quantification of relative luciferase activity in lung tissues. (K) H&E staining of lung tissues showing metastatic nodules. Scale bar: 100 µm. Data are presented as mean ± SD from eight mice per group (*n* = 8). Statistical significance was assessed using one‐way ANOVA for multiple group comparisons. *p < 0.05, **p < 0.01, ***p < 0.001.

Following the procedure outlined in **Figure** **7**A, we conducted in vivo studies to validate STAT3 reactivation's impact on α‐Hederin's anti‐OC efficacy in vivo. Figure 7B–D shows that Colivelin significantly counteracted the tumor growth inhibition by α‐Hederin. The histomorphology of vital organs showed no significant differences between mice treated with α‐Hederin alone and those treated with the combination of α‐Hederin and Colivelin (Figure 7E). Colivelin also reversed the α‐Hederin‐induced changes in protein levels of the tumor, including p‐STAT3 and Ki67 (Figure 7F,G). In tail vein metastasis models, Colivelin notably restored the lung metastasis nodules reduced by α‐Hederin (Figure 7H–K). These findings confirm that α‐Hederin inhibits the malignant phenotype of OC cells by targeting the JAK/STAT3 signaling pathway. These results supported α‐Hederin suppressed the malignant phenotype of OC cells through inhibition of the JAK/STAT3 pathway.

### 2.7 α‐Hederin Synergizes with CDDP to Suppress OC Progress and Overcome Chemoresistance

**Figure 8:**
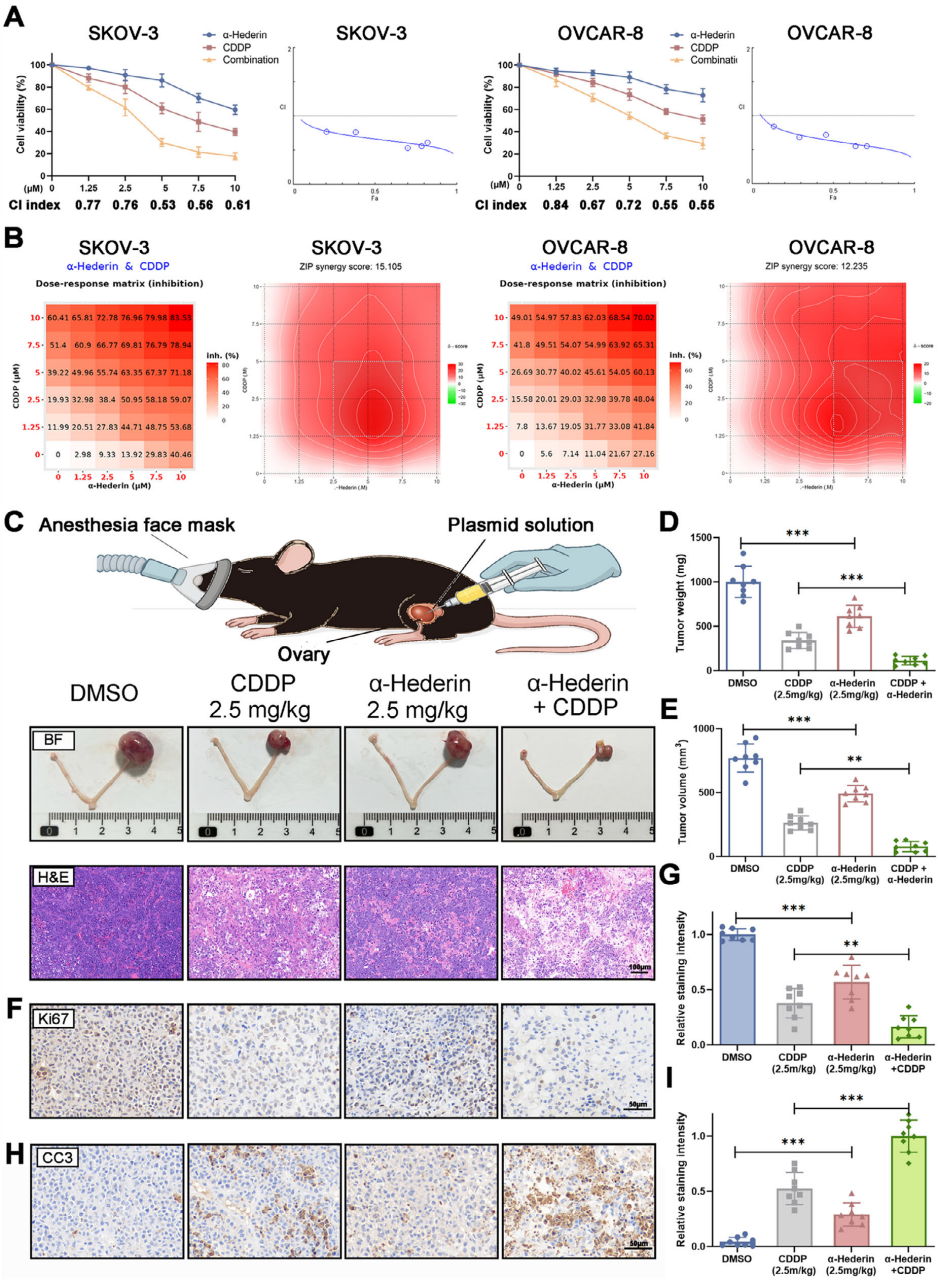
α‐Hederin synergizes with CDDP to suppress OC progression and enhance chemotherapy efficacy. (A) Cell viability of SKOV‐3 and OVCAR‐8 cells following treatment with α‐Hederin, CDDP, or their combination for 24 h, measured by CCK‐8 assay. CI values were calculated using CompuSyn. (B) Synergy analysis of α‐Hederin and CDDP using SynergyFinder. Left: dose‐response inhibition matrices; right: ZIP synergy score contour plots for SKOV‐3 and OVCAR‐8 cells. (C) Representative gross tumor images and H&E‐stained sections from SKOV‐3 orthotopic xenografts treated with DMSO, CDDP (2.5 mg kg^−1^), α‐Hederin (2.5 mg kg^−1^), or the combination. Scale bar (H&E): 100 µm. (D,E) Quantification of tumor weight (D) and volume (E) at the experimental endpoint. (F) IHC staining for Ki67. Scale bar: 50 µm. (G) Quantification of staining intensity from (F). (H) IHC staining for CC3. Scale bar: 50 µm. (I) Quantification of staining intensity from (H). Data are presented as mean ± SD from eight mice per group (*n* = 8). Statistical significance was assessed using one‐way ANOVA for multiple group comparisons. *p < 0.05, **p < 0.01, ***p < 0.001.

To evaluate the potential of α‐Hederin to enhance CDDP efficacy, we conducted combinatorial treatment experiments in OC cell lines SKOV‐3 and OVCAR‐8. As shown in **Figure** **8**A, α‐Hederin and CDDP co‐treatment significantly reduced cell viability compared to either monotherapy. Combination index (CI) values, calculated using CompuSyn, were consistently <1 across a range of concentrations, indicating strong synergism. This synergy was further confirmed via SynergyFinder analysis, where ZIP synergy scores exceeded 10 in both cell lines (Figure 8B), suggesting a robust and consistent synergistic interaction between α‐Hederin and CDDP.

To validate these findings in vivo, we employed an orthotopic OC xenograft mouse model. Mice treated with the α‐Hederin and CDDP combination exhibited a marked reduction in tumor size and weight compared to either agent alone (Figure 8C–E), indicating enhanced anti‐tumor efficacy. Histological analysis via H&E staining revealed increased necrotic regions in tumors from the combination group, further supporting the augmented therapeutic effect. IHC staining demonstrated that the combination therapy significantly reduced Ki67‐positive proliferative cells (Figure 8F,G) and elevated cleaved caspase‐3 (CC3)‐positive apoptotic cells (Figure 8H,I), suggesting that α‐Hederin enhances CDDP‐induced apoptosis while suppressing tumor cell proliferation. Additionally, to explore whether α‐Hederin can overcome platinum resistance, we established CDDP‐resistant OC cell lines SKOV‐3/CDDP and OVCAR‐8/CDDP by gradually exposing parental cells to increasing concentrations of CDDP (Table S3, Supporting Information). As summarized in Table S3 (Supporting Information), these resistant sublines displayed markedly elevated IC₅₀ values compared to their parental counterparts, with RI of 4.64 and 4.05, respectively, confirming the acquisition of a robust chemoresistant phenotype. To assess whether α‐Hederin can reverse this resistance, SKOV‐3/CDDP and OVCAR‐8/CDDP cells were pretreated with a low dose of α‐Hederin (2.5 µm) for 2 h, followed by CDDP exposure. As shown in Figure S7A (Supporting Information), α‐Hederin significantly enhanced CDDP‐induced cytotoxicity across all concentrations in both resistant cell lines. Colony formation assays further confirmed that α‐Hederin restored CDDP sensitivity, as combination‐treated groups exhibited a significant reduction in colony formation compared to either treatment alone (Figure S7B,C, Supporting Information). Collectively, these findings provide compelling preclinical evidence that α‐Hederin not only synergizes with CDDP to inhibit OC progress, but also effectively restores CDDP sensitivity in resistant cells, highlighting its potential as a chemosensitizing agent in platinum‐based therapy.

## 3 Discussion

Ovarian cancer (OC) remains a significant clinical challenge, often diagnosed at advanced stages with limited treatment options and a poor 5‐year survival rate.^[^ ^26^ ^]^ Standard chemotherapeutic agents, such as paclitaxel and carboplatin, initially induce remission but are ultimately undermined by high recurrence rates and acquired chemoresistance.^[^ ^27^, ^28^ ^]^ The severe side effects associated with long‐term chemotherapy further compromise therapeutic efficacy and patient quality of life. These clinical limitations underscore the urgent need for novel, effective, and low‐toxicity therapeutic agents.

Natural compounds have gained attention as potential anti‐cancer therapeutics due to their broad pharmacological activities and favorable safety profiles.^[^ ^29^, ^30^ ^]^ In this context, α‐Hederin, a pentacyclic triterpenoid saponin, emerges as a promising candidate. Our study systematically investigated its anti‐OC activity and mechanistic basis through a combination of virtual screening, molecular docking, kinase assays, and both in vitro and in vivo functional validations. Compared to currently available JAK inhibitors, such as Ruxolitinib, Baricitinib, and Fedratinib, α‐Hederin offers several unique advantages: i) it simultaneously inhibits both JAK1 and JAK2 enzymatic activity with high selectivity, which prevents compensatory activation mechanisms seen with single‐target agents; ii) it is a naturally derived compound with a favorable toxicity profile, as demonstrated in our in vivo models, where no myelosuppression, hepatotoxicity, or significant weight loss was observed, in contrast to the adverse effects often associated with synthetic JAK inhibitors; iii) it exerts multifaceted anti‐cancer effects, including suppression of EMT, induction of G0/G1 phase cell cycle arrest, inhibition of cell migration and invasion, collectively contributing to its broad anti‐tumor activity. Additionally, α‐Hederin significantly enhances the efficacy of CDDP, indicating its potential for combination therapy and synthetic lethality‐based approaches in OC.

Our results demonstrate that α‐Hederin selectively targets OC cells while sparing normal ovarian epithelial cells (IOSE‐80), highlighting its therapeutic safety window. This specificity likely arises from the unique molecular characteristics of cancer cells, such as abnormal signaling pathways and dysregulated cell cycle progression.^[^ ^31^ ^]^ Our study also revealed that α‐Hederin suppresses OC progression by inhibiting cell migration and invasion. EMT is a crucial biological process in cancer progression and metastasis, regulated by complex signaling pathways.^[^ ^32^ ^]^ In OC, the JAK/STAT3 signaling pathway is frequently and constitutively activated, particularly in high‐grade serous OC.^[^ ^33^, ^34^, ^35^ ^]^ STAT3, among the STAT protein family, is the most consistently upregulated in OC, as supported by our transcriptomic analysis (Figure 1D; Figure S1B, Supporting Information) and by previous studies showing its correlation with poor prognosis and aggressive tumor phenotypes.^[^ ^36^ ^]^ This oncogenic dominance of STAT3 underpins our mechanistic focus. Due to its central role in ovarian tumorigenesis and constraints on experimental resources, we initially concentrated on the JAK/STAT3 axis. Nevertheless, investigating the effects of α‐Hederin on other STAT isoforms, such as STAT1 or STAT5, would be valuable in future studies, especially given that JAK1 and JAK2 can phosphorylate multiple STATs under specific conditions. Mechanistically, α‐Hederin inhibits the JAK/STAT3 signaling axis, downregulates STAT3‐regulated oncogenic genes (MYC, CCND1, BIRC5, TWIST1),^[^ ^37^, ^38^ ^]^ and blocks STAT3 phosphorylation and nuclear translocation. These findings were validated by STAT3 rescue assays using Colivelin, which partially reversed α‐Hederin‐induced suppression of proliferation, migration, and EMT.

Molecular docking confirmed α‐Hederin's high binding affinity to the ATP‐binding JH1 domain of both JAK1 and JAK2. In vitro kinase inhibition and DARTS assays further substantiated its direct enzymatic targeting. This distinguishes α‐Hederin from previously studied agents which only demonstrated its impact on IL‐6‐induced STAT3 activation in colon cancer, without identifying its direct molecular targets or effects in OC.^[^ ^39^, ^40^ ^]^ Furthermore, our study employed three independent in vivo models—a subcutaneous xenograft model, a tail vein‐injection lung metastasis model, and an orthotopic OC model—to comprehensively validate the anti‐tumor efficacy and safety of α‐Hederin. These data provide strong preclinical support for clinical translation.

Despite promising results, our study has several limitations that must be addressed in future work. First, although we provide evidence for JAK1/JAK2‐STAT3 pathway inhibition by α‐Hederin in vitro and in vivo, the long‐term pharmacokinetic profile, metabolic stability, and oral bioavailability of α‐Hederin remain poorly defined. Comprehensive ADME‐Tox (Absorption, Distribution, Metabolism, Excretion, and Toxicology) profiling and dose‐escalation safety studies are required in multiple animal models to support investigational new drug (IND) applications. Second, although we have evaluated the therapeutic efficacy of α‐Hederin in an orthotopic OC model with an intact immune system, further studies are warranted to elucidate its interactions with the tumor immune microenvironment, as well as to assess immune‐related toxicities, particularly in combination with CDDP. Third, although our study primarily focused on the JAK/STAT3 pathway, broader target validation such as kinase panel screening and genetic dependency assays is necessary to definitively exclude potential off‐target effects.

Compared to other natural compounds with anticancer potential, such as Curcumin, Resveratrol, and Berberine, α‐Hederin demonstrates a distinct mechanism of action by functioning as a dual JAK1/JAK2 inhibitor with selective activity against cancer cells. Notably, its synergistic effects with platinum‐based chemotherapy and low toxicity in normal epithelial cells may offer a therapeutic window not commonly observed with other natural agents. However, to establish α‐Hederin as a viable clinical candidate, GMP‐grade formulation development, delivery optimization (e.g., nanoparticles, prodrugs), and early‐phase toxicity trials in primates are important next steps.

**Figure 9:**
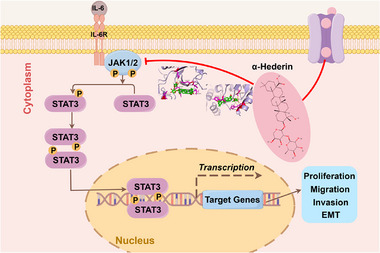
Proposed schematic model of the anti‐tumor mechanism of α‐Hederin in OC. α‐Hederin directly binds to JAK1/2 kinases, inhibiting JAK‐mediated phosphorylation of STAT3. This blocks STAT3 activation and its subsequent nuclear translocation, leading to downregulation of target gene transcription involved in proliferation, migration, invasion, and EMT in OC cells.

In conclusion, α‐Hederin represents a novel dual JAK1/JAK2 inhibitor that suppresses OC progression via blockade of the JAK/STAT3 pathway (**Figure** **9**), while offering superior safety and therapeutic advantages over existing synthetic inhibitors. Its ability to enhance the activity of CDDP further supports its development as a combination therapy agent. These findings lay a solid foundation for future clinical evaluation of α‐Hederin in OC treatment.

## 4 Experimental Section

### Cell Culture and Reagents

Human OC cells SKOV‐3 and OVCAR‐8 were purchased from Pricella Biotechnology (Wuhan, China). Human normal ovarian epithelial cell line IOSE‐80 was purchased from Beijing Bowers Type Culture Collection. SKOV‐3 cells were cultured in McCoy's 5A medium (Pricella, Wuhan, China) containing 10% fetal bovine serum (FBS, Cellmax, China) and 1% penicillin‐streptomycin. OVCAR‐8 and IOSE‐80 cells were cultured in RMPI 1640 medium (Pricella) containing 10% fetal bovine serum and 1% penicillin‐streptomycin. All the cells were cultured in the cell culture dishes/plates which were obtained from Bioland (China). All cells were cultured in an incubator at 37°C with 5% CO2. All experiments were repeated at least three times to ensure the reliability of the results. CDDP‐resistant SKOV‐3 and OVCAR‐8 cell lines were established by exposing parental cells to stepwise increasing concentrations of CDDP. Cells were initially treated with 0.5 µm CDDP for 72 h. Surviving cells were allowed to recover, followed by incremental dose escalation by 0.5 µm every 7–10 days until reaching a final concentration of 10 µm. At each step, only stably growing cells were used for subsequent passages. Resistant sublines, designated as SKOV‐3/CDDP and OVCAR‐8/CDDP, were maintained in medium containing 1 µm CDDP to preserve the resistance phenotype and cultured in drug‐free medium for 72 h before experiments. To evaluate the level of acquired resistance, the resistance index (RI) was calculated as the ratio of the IC₅₀ value of the resistant cell line to that of its corresponding parental line, according to the following formula: RI═​IC_50, resistant_ / IC_50, parental_.

α‐Hederin (purity ≥99.8%), dimethyl sulfoxide (DMSO), the positive control CDDP, and the STAT3 agonist (Colivelin) were purchased from MedChemExpress (Shanghai, China). α‐Hederin was dissolved in DMSO to prepare a 20 mm stock solution and stored at −20 °C. The recombinant JAK1, JAK2, and STAT3 were also purchased from MedChemExpress (Shanghai, China).

### Antibodies

The antibodies utilized in this study include GAPDH, E‐cadherin, N‐cadherin, Ki67, and Snail, all sourced from Proteintech (Wuhan, China). Antibodies for Cyclin D1 and CDK4 were purchased from ABclonal Technology (WuHan, China). Antibodies for JAK1, p‐JAK1, SRC, p‐SRC, JAK2, p‐JAK2, STAT3, and p‐STAT3 (Y705) were obtained from Cell Signaling Technology (Danvers, MA, USA). Additionally, Vimentin and p53 antibodies were acquired from Affinity Biosciences (Cincinnati, OH, USA). Cleaved‐Caspase 3, JAK3 and p‐JAK3 was purchased from Bioss (USA).

### Cell Viability Assay

Cell viability was detected using the Cell Counting Kit‐8 (CCK‐8) reagent (Biosharp, Hefei, China). Cells were seeded at a density of 8 × 10^3 cells per well in 96‐well plates. After drug treatment, 10 µl of CCK‐8 reagent (Biosharp) was added to each well and incubated at 37 °C for 1–2 h. Subsequently, the optical density (OD) at 450 nm was measured using a microplate reader (Perkin Elmer, Waltham, MA, USA).

### Bioinformatics Analysis

Bioinformatics analysis utilized TCGA and GTEx datasets to identify differentially expressed genes (DEGs). The analysis pipeline included statistical normalization (DESeq2), pathway enrichment using KEGG, and visualization via Cytoscape, ensuring reproducibility and clarity of the findings. Single‐cell gene expression data and associated cell annotations for OC samples were obtained from a recent study, accessible through the Gene Expression Omnibus (GEO) under accession number GSE165897. This dataset includes 11 high‐grade serous ovarian cancer (HGSOC) patients who underwent neoadjuvant chemotherapy (NACT), with paired pre‐ and post‐treatment samples profiled using scRNA‐seq. The dataset was analyzed as provided, without additional quality control, as QC measures were already applied in the original study. The Seurat package (version:5.1.0, https://github.com/satijalab/seurat) was utilized to process the data for downstream analysis. Using the AddMetaData function, metadata was integrated into the dataset, and RunUMAP was employed for dimensionality reduction and visualization.

### Virtual Screening

The virtual screening process was conducted using Schrödinger Maestro 12.8, starting with the 3D structures of human JAK1 (PDB ID: 6GGH) and JAK2 (PDB ID: 4AQC), which were downloaded from the PDB database and processed using the Protein Preparation Wizard module to optimize structures and add hydrogens. The Traditional Chinese Medicine Active Compound Library, containing 2908 compounds in 2D format, was prepared using the Schrödinger LigPrep module for hydrogen addition, energy optimization, and 3D conversion. Prepared compounds were imported into the Virtual Screening Workflow module for docking, where the Glide module was used to perform molecular docking by matching receptor and ligand molecules based on geometric and energy compatibility. Docking was conducted in a stepwise manner, beginning with high‐throughput virtual screening (HTVS) to filter compounds, followed by standard precision (SP) docking for refined scoring, and extra precision (XP) docking to identify top‐ranked compounds with detailed binding interactions. Post‐docking analysis confirmed the involvement of key residues in stable binding through hydrogen bonding and hydrophobic interactions, ensuring the selection of promising candidates for further validation.

### Network Pharmacology

The target information for α‐Hederin (Compound CID: 73296) was retrieved from both the PubChem database (https://pubchem.ncbi.nlm.nih.gov/) and the SwissTargetPrediction database (http://www.swisstargetprediction.ch/). After eliminating redundant genes, these targets were converted into gene names using the UniProt database (https://www.uniprot.org/). To identify potential targets related to OC, searches were conducted in the GeneCards (https://www.genecards.org/, relevance score>15.0), DisGeNET (https://www.disgenet.org/, score_gda≥0.1), and OMIM (https://omim.org/) databases, followed by the removal of duplicate genes.

Using the Venny 2.1 tool (https://bioinfogp.cnb.csic.es/tools/venny/), a Venn diagram was created to show the overlap between the targets of α‐Hederin and OC, highlighting common targets that may indicate potential therapeutic applications. These shared targets, focusing on Homo sapiens, were then used to construct a protein‐protein interaction (PPI) network via the STRING database, with a confidence score threshold of 0.4. The network visualization was carried out using Cytoscape 3.9.1, with targets organized by degree, depicted through gradient colors and sizes.

Kyoto Encyclopedia of Genes and Genomes (KEGG) pathway analysis was conducted using the DAVID platform (david.ncifcrf.gov/). These analyses provided valuable insights into the biological functions and pathways associated with the common targets, shedding light on the potential therapeutic mechanisms through which α‐Hederin may exert its effects in the treatment of OC.

### Microscale Thermophoresis (MST)

The binding affinity of α‐Hederin with recombinant JAK1 (MCE, HY‐P700583) and JAK2 (MCE, HY‐P7989) was analyzed using the Microscale thermophoresis Monolith NT.115 system (Nanotemper Technologies) from Core Facility of Medical Research Institute at Wuhan University. For protein‐ligand affinity testing, recombinant JAK1 and JAK2 proteins were diluted in PBS‐T, and adjusted to a final concentration of 200 nm. Labeling was performed using the RED‐Tris‐NTA Protein Labeling Kit (MO L018) according to the manufacturer's guidelines. Compounds (stock solutions 20 mm) were serially diluted and incubated with labeled proteins for the MST assay. The samples were loaded into premium capillaries, and thermophoresis was measured under specified power conditions. Kd values were calculated using Nanotemper analysis software, demonstrating significant binding of α‐Hederin to both JAK1 and JAK2, underscoring its potential as a therapeutic agent targeting these kinases.

### 5‐Ethynyl‐2’‐Deoxyuridine (EdU) Assay

First, cells were seeded at a density of 2.5 × 10^5 in 12‐well plates and cultured in a 37 °C, 5% CO₂ incubator. After drug treatment, 10 µm EdU reagent (Beyotime, Shanghai, China) was added to the medium and incubated for 2 h to allow incorporation of EdU during DNA synthesis. After incubation, the medium was removed and the cells were fixed with 4% paraformaldehyde (Biosharp) at room temperature for 15 min. Then, the cells were permeabilized with 0.5% Triton X‐100 (Biosharp) at room temperature for 10 min. After permeabilization, the reaction solution was prepared according to the manufacturer's instructions and incubated in the dark for 30 min. Finally, the cell nuclei were stained with DAPI, and the EdU‐labeled cells were observed under a fluorescence microscope (BX51, Olympus, Tokyo, Japan).

### Colony Formation Assay

The colony formation assay was used to assess cell proliferation and colony‐forming ability. First, cells were seeded at a density of 1 × 10^3 in six‐well plates to allow single cells to grow into colonies independently. After drug treatment, the cells were incubated for another 14 days, with the fresh medium changed regularly. At the end of the incubation, the cells were fixed with 4% paraformaldehyde (Biosharp) for 15 min. Then, the cells were stained with 0.1% crystal violet (Biosharp) for 30 min. After staining, excess dye was washed off with tap water, and after the plates dried, photographs were taken and the number of colonies in each plate was counted to evaluate the colony‐forming ability of the cells.

### Cell Cycle Analysis

First, the cells to be tested were collected and washed with PBS, then fixed overnight with 70% cold ethanol to ensure cell membrane permeability. After fixation, the cells were treated with PBS solution containing RNase A (100 µg mL^−1^) to remove RNA, then stained with 50 µg mL^−1^ propidium iodide (PI, Elabscience Biotechnology Co., Ltd., China) in the dark for 30 min to bind DNA with the fluorescent dye. The fluorescence intensity of the cells was then detected using a CytoFLEX flow cytometer (Beckman Coulter, USA). Finally, the cell cycle distribution was analyzed by FlowJo software (TreeStar, USA).

### Scratch Assay

The Scratch assay was a method used to evaluate cell migration ability. First, cells were cultured in 6‐well plates until ≈90% confluency, then a sterile pipette tip was used to vertically scratch a straight line in the center of the cell layer to create a wound. Next, the plate was gently washed with PBS to remove detached cells and debris, then the cells were treated with appropriate drug concentrations. The plate was then placed under a microscope to capture images of the scratch at 0 h, and the cells were continuously cultured with images taken at regular intervals to monitor wound healing. At the end of the experiment, cell migration ability was evaluated by measuring the reduction in the scratch area.

### Transwell Assay

The Transwell migration and invasion assays were methods used to evaluate cell migration and invasion abilities. First, cells were suspended in the serum‐free medium and seeded into the upper chamber of a Transwell insert (NEST Biotechnology, China). For migration assays, no Matrigel (BD Biosciences, USA) was added. The bottom of the upper chamber was precoated with Matrigel to simulate the extracellular matrix for invasion assays. Then, 600 µL of complete medium containing 15% FBS was added to the lower chamber to induce cell migration or invasion. After 24 h of incubation, non‐migrated or non‐invaded cells in the upper chamber were gently removed with a cotton swab. Next, cells were fixed with 4% paraformaldehyde and stained with 0.1% crystal violet. Finally, the stained cells on the bottom membrane of the lower chamber were observed and counted under a microscope to evaluate cell migration and invasion abilities.

### Immunofluorescence (IF)

First, cells were fixed with 4% paraformaldehyde for 15 min. Next, cells were permeabilized with 0.3% Triton X‐100 (excluding membrane proteins) to increase cell membrane permeability. Then, the cells were blocked with 5% BSA at room temperature for 30 min. Next, primary antibodies were added and incubated overnight at 4 °C in a humidified chamber. After that, fluorescently labeled secondary antibodies were added and incubated for 30 min in the dark. F‐actin was labeled by YF^®^594 ‐ Phalloidin (UElandy, Suzhou, China). Then, DAPI was added to stain the nuclei and incubated for 10 min in the dark. Finally, the cells were mounted with an anti‐fade mounting medium and observed under a fluorescence microscope to evaluate the expression and localization of target antigens.

### Immunohistochemistry (IHC)

First, tissue samples were fixed in formalin, dehydrated, embedded, and sectioned, with sections mounted on slides. Next, antigen retrieval was performed in a microwave using sodium citrate buffer to expose antigen sites. Then, sections were treated with 3% hydrogen peroxide to inhibit endogenous peroxidase activity and blocked with serum to prevent non‐specific binding. Next, primary antibodies were added and incubated overnight in a humid chamber, followed by the addition of secondary antibodies and incubation. Streptavidin‐peroxidase complex was then added for labeling. The DAB substrate was used for color development until a color signal appeared, and hematoxylin was used for counterstaining the nuclei. Finally, the sections were dehydrated through a graded alcohol series, cleared, and mounted with neutral balsam. The staining results were observed under a microscope to evaluate the expression and localization of the target antigens.

### Western Blot Analysis

First, protein samples were separated by SDS‐PAGE electrophoresis and transferred onto a polyvinylidene fluoride membrane. Next, the membrane was blocked with 5% skim milk for 1 h. Then, specific primary antibodies were added and incubated overnight at 4 °C, followed by washing the membrane four times with TBST, each for 5 min. Next, appropriate HRP‐conjugated secondary antibodies were added and incubated for 1 h at room temperature, followed by washing the membrane four times with TBST. Finally, the Sparkjade ECL plus (Shandong Sparkjade Biotechnology Co., Ltd.) was used for color developmentas recommended by the manufacturer. And protein bands were detected using an ECL imager (ChemiDoc, Bio‐Rad, Hercules, CA, USA). Quantitative analysis was performed using ImageJ software.

### In Vivo Xenograft Model

Forty‐two female nude mice aged 4–6 weeks (19–21g) were purchased from Weitonglihua Biotechnology (Beijing, China). SKOV‐3 cells in culture were collected, resuspended in PBS, and prepared into a single‐cell suspension (1.0 × 10^7/mL), then subcutaneously injected into each nude mouse (0.2 mL per mouse). When the average tumor volume reached 50 mm^3^, the mice were randomly divided into seven groups (6 mice/group) and intraperitoneally administered DMSO, α‐Hederin (2.5 mg kg^−1^), α‐Hederin (5 mg kg^−1^), CDDP (2.5 mg kg^−1^), or DMSO, α‐Hederin (5 mg kg^−1^), Colivelin (1 mg kg^−1^) + α‐Hederin (5 mg kg^−1^) every 3 days. The body weight and tumor size of the nude mice were measured every 3 days. The tumor volume (TV) was calculated using the formula: TV (mm^3^) = 0.5 × d^2 × D, where d represents the short diameter and D represents the long diameter. After 21 days, the nude mice were sacrificed, the tumor tissues were dissected, and the tumor weight was recorded. Additionally, kidney and liver functions were assessed by serum levels of Urea, creatinine (Crea), aspartate aminotransferase (AST), and alanine aminotransferase (ALT). The tissue sections were stained with H&E staining kit (Beijing Solarbio Science & Technology Co., Ltd.) based on the manufacturer's instructions.

All animal experiments were conducted in accordance with the NIH Guide for the Care and Use of Laboratory Animals (NIH Publication No. 80‐23, revised 1978) and received approval from the Laboratory Animal Welfare and Ethics Committee of Renmin Hospital (IACUC Issue No. WDRM20240906B).

### Lung Metastatic Model

An SKOV‐3 cell line carrying the Luc luciferase reporter gene (SKOV‐3‐Luc) was first constructed. Then, 1 × 10^6 SKOV‐3‐Luc cells were injected into the tail vein of nude mice to establish a lung metastasis model. Subsequently, the bioluminescence of the tumors was detected using an optical imaging system (IVIS spectrum CT, Perkin Elmer, Waltham, MA, USA). At the end of the experiment, the formation and development of metastatic tumors were assessed by dissecting and histologically analyzing lung tissues.

### Orthotopic OC Model

An orthotopic OC model was established in 8‐week‐old female C57BL/6L mice via intra‐ovarian injection of oncogenic plasmids. Mice were anesthetized using 2–3% isoflurane administered via an inhalation system. After confirming deep anesthesia, mice were placed in the prone position, and the dorsal skin was sterilized with 70% ethanol. A small incision was made in the lower back to access the retroperitoneal space. Through gentle blunt dissection, the ovary was exposed using curved forceps, taking care to avoid damaging the surrounding tissues. A plasmid mixture containing pT3‐AKT, pT3‐MYC, and pCAG‐SB100 (transposase system) was prepared in sterile saline and loaded into a 30‐gauge insulin syringe. After injection, the site was gently compressed with a sterile cotton swab for several seconds to minimize leakage. The ovary was then repositioned into the peritoneal cavity, and the abdominal muscle and skin layers were sutured separately using absorbable sutures. Postoperative analgesia was provided by subcutaneous injection of meloxicam (5 mg kg^−1^) immediately after surgery and once daily for the following 2 days. Mice were monitored during recovery from anesthesia and housed in clean cages with ad libitum access to food and water. The orthotopic injection procedure was technically supported by Shouzheng Hongyao (Wuhan) Biotechnology Co., Ltd.

### Kinase Activity Assay

The kinase activity of JAK1 (Signal Chem, Canada) and JAK2 (Signal Chem, Canada) was measured by ADP‐Glo™ Kinase Assay (Promega, V6930) based on the manufacturer's instruction. An ADP‐Glo™ kinase assay was performed to assess kinase activity by first depleting unconsumed ATP, converting ADP to ATP, and then measuring the ATP consumption. In brief, a kinase buffer containing active kinase and 100 µm ATP, along with DMSO or α‐Hederin, was incubated at 25°C for 30 min. Following this, ADP‐Glo™ reagent was added, and the mixture was incubated for an additional 40 min at 25°C. The kinase activity was then quantified using a microplate reader (PerkinElmer, Waltham, MA, USA).

### ATP Competition Assay

Recombinant JAK1 and JAK2 obtained from Signal Chem (Canada), were incubated with specified concentrations of α‐Hederin or ATP (New England Biolabs, N0440S) in a binding buffer (comprising 50 mM Tris pH 7.4, 150 mm NaCl, 1 mm EDTA, 6 mm sodium deoxycholate, 1% NP‐40, 1 mm PMSF, and a protease inhibitor cocktail) at 4 °C for an hour. The mixture was then rotated overnight at 4 °C with either ATP‐conjugated Sepharose beads (GE Healthcare) or α‐Hederin‐conjugated Sepharose beads. Following this, the beads were washed five times with the binding buffer, and the amount of JAK bound to the beads was analyzed via Western blot.

### Drug Affinity Responsive Target Stability (DARTS)

Cells were washed with PBS and scraped off in M‐PER buffer (Thermo Scientific, 78501) with PMSF (Servicebio, G2008) and protease inhibitors (MCE, HY‐K0010) on ice. The supernatant was transferred to a fresh tube, where TNC buffer (50 mm Tris‐HCl pH 8.0, 50 mm NaCl, 10 mm CaCl2) was added. The cell lysate was then incubated with specified concentrations of α‐Hederin for 30 min on ice, followed by a 30‐min incubation at 25°C. Proteolytic digestion was carried out using 50 µg mL^−1^ Pronase (Roche, 10165921001) at room temperature for 30 min. To halt the digestion, an SDS loading buffer was added, and the samples were boiled for 5 min. The samples were then subjected to SDS‐PAGE and Western blot analysis.

### In Vitro Kinase Assay

Following standardized procedures, an in vitro kinase assay was performed to investigate the influence of α‐Hederin on JAK1 and JAK2 enzymatic activity. His‐tagged STAT3 was expressed in *E. coli* cells and subsequently purified using Ni‐NTA beads, serving as the substrate in kinase assays. Active human JAK1 and JAK2 kinase, obtained from Signal Chem (Canada), was diluted to a final concentration of 0.1 µg mL^−1^ in Kinase Dilution Buffer III (Signal Chem, K23‐09). This was then combined with 3 µg of STAT3 and 5 µl of ATP (New England Biolabs, N0440S). The assays included varying concentrations of α‐Hederin (5 µm and 10 µm) and were carried out at 30 °C for 30 min. The kinase reactions were terminated by adding 2× SDS loading buffer and heating the mixture at 95 °C for 5 min. To confirm the phosphorylation of STAT3 at Tyr705 by JAK2, the Western blot was performed using an anti‐phosphorylated STAT3^Y705^ antibody.

### Statistical Analysis

All in vitro experiments were conducted in triplicate, and results were expressed as mean ± standard deviation (SD). Data were analyzed using GraphPad Prism software (version 8.0, GraphPad Software, San Diego, CA, USA). Comparisons between the two groups were made using the Student's *t*‐test. For comparisons among multiple groups, the one‐way analysis of variance (ANOVA) was employed. *p*‐values less than 0.05 were considered statistically significant. All figures were generated using GraphPad Prism, and significant differences were indicated as follows: p* < 0.05, p** < 0.01, and p*** < 0.001, ns: not significant.

### Ethical Approval Statement

The Laboratory Animal Welfare and Ethics Committee of Renmin Hospital of Wuhan University reviewed and approved the animal procedures (IACUC Issue No. WDRM20240906B). All animal experiments were conducted following the NIH Guide for the Care and Use of Laboratory Animals (NIH Publication No. 80‐23; revised 1978).

## Conflict of Interest

The authors declare no conflict of interest.

## Author Contributions

J.W., P.H., and C.L. contributed equally to this work. J.W. contributed to conceptualization, methodology, and original draft preparation. P.H. was responsible for conceptualization, data curation, and software. C.L. contributed to conceptualization, formal analysis, and validation. X.C., Y.T., and R.Q. performed visualization and investigation. T.Y. and Z.L. provided supervision and acquired funding. Y.Z. contributed to validation, and writing – review and editing. Z.Y. supervised the study, acquired funding, and contributed to writing – review and editing.

## Supporting information



Supporting Information

## Data Availability

The data that support the findings of this study are available from the corresponding author upon reasonable request.

## References

[1] F. Bray , M. Laversanne , H. Sung , J. Ferlay , R. L. Siegel , I. Soerjomataram , A. Jemal , CA Cancer J. Clin. 2024, 74, 229. 38572751 10.3322/caac.21834

[2] D. K. Armstrong , R. D. Alvarez , J. N. Bakkum‐Gamez , L. Barroilhet , K. Behbakht , A. Berchuck , L.‐M. Chen , M. Cristea , M. DeRosa , E. L. Eisenhauer , D. M. Gershenson , H. J. Gray , R. Grisham , A. Hakam , A. Jain , A. Karam , G. E. Konecny , C. A. Leath , J. Liu , H. Mahdi , L. Martin , D. Matei , M. McHale , K. McLean , D. S. Miller , D. M. O'Malley , S. Percac‐Lima , E. Ratner , S. W. Remmenga , R. Vargas , et al., J. Natl. Compr. Cancer Network 2021, 19, 191. 10.6004/jnccn.2021.0007 33545690

[3] Y. Luo , Y. Xia , D. Liu , X. Li , H. Li , J. Liu , D. Zhou , Y. Dong , X. Li , Y. Qian , C. Xu , K. Tao , G. Li , W. Pan , Q. Zhong , X. Liu , S. Xu , Z. Wang , R. Liu , W. Zhang , W. Shan , T. Fang , S. Wang , Z. Peng , P. Jin , N. Jin , S. Shi , Y. Chen , M. Wang , X. Jiao , et al., Cell 2024, 187, 4905. 38971151 10.1016/j.cell.2024.06.013

[4] S. Banerjee , K. N. Moore , N. Colombo , G. Scambia , B.‐G. Kim , A. Oaknin , M. Friedlander , A. Lisyanskaya , A. Floquet , A. Leary , G. S. Sonke , C. Gourley , A. Oza , A. González‐Martín , C. Aghajanian , W. H. Bradley , E. Holmes , E. S. Lowe , P. DiSilvestro , Lancet Oncol. 2021, 22, 1721. 34715071 10.1016/S1470-2045(21)00531-3

[5] J. W. Zhu , P. Charkhchi , M. R. Akbari , Mol. Cancer 2022, 21, 114. 35545786 10.1186/s12943-022-01588-8 PMC9092780

[6] A. B. Nagaraj , M. Knarr , S. Sekhar , R. S. Connor , P. Joseph , O. Kovalenko , A. Fleming , A. Surti , E. Nurmemmedov , L. Beltrame , S. Marchini , M. Kahn , A. DiFeo , Cancer Res. 2021, 81, 2044. 33574092 10.1158/0008-5472.CAN-20-2041 PMC8137569

[7] J. Wang , Y. Sun , R. Wu , Adv. Biol. (Weinh.) 2025, 9, 2400093. 10.1002/adbi.202400093 39913127

[8] G. Gritsina , F. Xiao , S. W. O'Brien , R. Gabbasov , M. A. Maglaty , R.‐H. Xu , R. J. Thapa , Y. Zhou , E. Nicolas , S. Litwin , S. Balachandran , L. J. Sigal , D. Huszar , D. C. Connolly , Mol. Cancer Ther. 2015, 14, 1035. 25646015 10.1158/1535-7163.MCT-14-0800 PMC4394029

[9] M. A. Samad , I. Ahmad , A. Hasan , M. H. Alhashmi , A. Ayub , F. A. Al‐Abbasi , A. Kumer , S. Tabrez , MedComm 2025, 6, 70152. 10.1002/mco2.70152 PMC11955304 40166646

[10] H. Wang , Y. Fu , BMC Cancer 2021, 21, 871. 34330232 10.1186/s12885-021-08597-8 PMC8323274

[11] C. N. Harrison , R. Mesa , M. Talpaz , H. K. Al‐Ali , B. Xicoy , F. Passamonti , F. Palandri , G. Benevolo , A. M. Vannucchi , C. Mediavilla , A. Iurlo , I. Kim , S. Rose , P. Brown , C. Hernandez , J. Wang , J.‐J. Kiladjian , Lancet Haematol. 2024, 11, 729. 10.1016/S2352-3026(24)00212-6 39265613

[12] P. Bharathiraja , P. Yadav , A. Sajid , S. V. Ambudkar , N. R. Prasad , Drug Resist. Updates 2023, 71, 101004. 10.1016/j.drup.2023.101004 PMC10840887 37660590

[13] H. N. Pham , C. A. Tran , T. D. Trinh , N. L. Nguyen Thi , H. N. Tran Phan , V. N. Le , N. H. Le , V. T. Phung , J. Anal. Methods Chem. 2022, 2022, 1167265. 35979140 10.1155/2022/1167265 PMC9377918

[14] A. I. Gavrila , R. Tatia , A.‐M. Seciu‐Grama , I. Tarcomnicu , C. Negrea , I. Calinescu , C. Zalaru , L. Moldovan , A. D. Raiciu , I. Popa , Pharmaceuticals (Basel) 2022, 15, 1197. 36297309 10.3390/ph15101197 PMC9609769

[15] J. Li , D.‐D. Wu , J.‐X. Zhang , J. Wang , J.‐J. Ma , X. Hu , W.‐G. Dong , World J. Gastroenterol. 2018, 24, 1901. 29740205 10.3748/wjg.v24.i17.1901 PMC5937207

[16] J. Sun , Y. Feng , Y. Wang , Q. Ji , G. Cai , L. Shi , Y. Wang , Y. Huang , J. Zhang , Q. Li , Int. J. Oncol. 2019, 54, 1601. 30896843 10.3892/ijo.2019.4757 PMC6438428

[17] M. S. Butt , M. T. Sultan , Crit. Rev. Food Sci. Nutr. 2010, 50, 654. 20694927 10.1080/10408390902768797

[18] H. Gumushan‐Aktas , S. Altun , Oncol. Lett. 2016, 12, 2985. 27698887 10.3892/ol.2016.4941 PMC5038493

[19] D. Sun , W. Shen , F. Zhang , H. Fan , J. Tan , L. Li , C. Xu , H. Zhang , Y. Yang , H. Cheng , Biomed Res. Int. 2018, 2018, 2548378. 30363706 10.1155/2018/2548378 PMC6180961

[20] M. J. Goldman , B. Craft , M. Hastie , K. Repečka , F. McDade , A. Kamath , A. Banerjee , Y. Luo , D. Rogers , A. N. Brooks , J. Zhu , D. Haussler , Nat. Biotechnol. 2020, 38, 675. 32444850 10.1038/s41587-020-0546-8 PMC7386072

[21] B. Izar , I. Tirosh , E. H. Stover , I. Wakiro , M. S. Cuoco , I. Alter , C. Rodman , R. Leeson , M.‐J. Su , P. Shah , M. Iwanicki , S. R. Walker , A. Kanodia , J. C. Melms , S. Mei , J.‐R. Lin , C. B. M. Porter , M. Slyper , J. Waldman , L. Jerby‐Arnon , O. Ashenberg , T. J. Brinker , C. Mills , M. Rogava , S. Vigneau , P. K. Sorger , L. A. Garraway , P. A. Konstantinopoulos , J. F. Liu , U. Matulonis , et al., Nat. Med. 2020, 26, 1271. 32572264 10.1038/s41591-020-0926-0 PMC7723336

[22] P. S. Thilakasiri , R. S. Dmello , T. L. Nero , M. W. Parker , M. Ernst , A. L. Chand , Semin. Cancer Biol. 2021, 68, 31. 31711994 10.1016/j.semcancer.2019.09.022

[23] K. Zhang , E. P. Erkan , S. Jamalzadeh , J. Dai , N. Andersson , K. Kaipio , T. Lamminen , N. Mansuri , K. Huhtinen , O. Carpén , S. Hietanen , J. Oikkonen , J. Hynninen , A. Virtanen , A. Häkkinen , S. Hautaniemi , A. Vähärautio , Sci. Adv. 2022, 8, abm1831. 10.1126/sciadv.abm1831 PMC8865800 35196078

[24] Q. Hu , Y.‐C. Hou , J. Huang , J.‐Y. Fang , H. Xiong , J. Exp. Clin. Cancer Res. 2017, 36, 50. 28399898 10.1186/s13046-017-0526-0 PMC5387201

[25] G. Xiong , R. L. Stewart , J. Chen , T. Gao , T. L. Scott , L. M. Samayoa , K. O'Connor , A. N. Lane , R. Xu , Nat. Commun. 2018, 9, 4456. 30367042 10.1038/s41467-018-06893-9 PMC6203834

[26] P. A. Konstantinopoulos , U. A. Matulonis , Nat. Cancer 2023, 4, 1239. 37653142 10.1038/s43018-023-00617-9

[27] A. R. Clamp , E. C. James , I. A. McNeish , A. Dean , J.‐W. Kim , D. M. O'Donnell , J. Hook , C. Coyle , S. Blagden , J. D. Brenton , R. Naik , T. Perren , S. Sundar , A. D. Cook , G. S. Gopalakrishnan , H. Gabra , R. Lord , G. Dark , H. M. Earl , M. Hall , S. Banerjee , R. M. Glasspool , R. Jones , S. Williams , A. M. Swart , S. Stenning , M. Parmar , R. Kaplan , J. A. Ledermann , Lancet 2019, 394, 2084. 31791688 10.1016/S0140-6736(19)32259-7 PMC6902268

[28] F. Di Meo , S. Filosa , M. Madonna , G. Giello , A. Di Pardo , V. Maglione , A. Baldi , S. Crispi , J. Exp. Clin. Cancer Res. 2019, 38, 360. 31419989 10.1186/s13046-019-1368-8 PMC6698046

[29] Y.‐H. Wang , R.‐R. Zhang , Y. Yin , G.‐F. Tan , G.‐L. Wang , H. Liu , J. Zhuang , J. Zhang , F.‐Y. Zhuang , A.‐S. Xiong , J. Adv. Res. 2022, 46, 31. 35753652 10.1016/j.jare.2022.06.010 PMC10105081

[30] H. Xu , H. Zhao , C. Ding , D. Jiang , Z. Zhao , Y. Li , X. Ding , J. Gao , H. Zhou , C. Luo , G. Chen , A. Zhang , Y. Xu , H. Zhang , Signal Transduct. Target Ther. 2023, 8, 51. 36732502 10.1038/s41392-022-01231-4 PMC9895061

[31] H. O. Caglar , C. Biray Avci , Mol. Biol. Rep. 2020, 47, 3065. 32112300 10.1007/s11033-020-05341-6

[32] J. Yang , P. Antin , G. Berx , C. Blanpain , T. Brabletz , M. Bronner , K. Campbell , A. Cano , J. Casanova , G. Christofori , S. Dedhar , R. Derynck , H. L. Ford , J. Fuxe , A. García de Herreros , G. J. Goodall , A.‐K. Hadjantonakis , R. Y. J. Huang , C. Kalcheim , R. Kalluri , Y. Kang , Y. Khew‐Goodall , H. Levine , J. Liu , G. D. Longmore , S. A. Mani , J. Massagué , R. Mayor , D. McClay , K. E. Mostov , et al., Nat. Rev. Mol. Cell Biol. 2020, 21, 341. 32300252 10.1038/s41580-020-0237-9 PMC7250738

[33] I. A. Myles , E. D. Anderson , N. J. Earland , K. A. Zarember , I. Sastalla , K. W. Williams , P. Gough , I. N. Moore , S. Ganesan , C. J. Fowler , A. Laurence , M. Garofalo , D. B. Kuhns , M. D. Kieh , A. Saleem , P. A. Welch , D. A. Darnell , J. I. Gallin , A. F. Freeman , S. M. Holland , S. K. Datta , J. Clin. Invest. 2018, 128, 3595. 30035749 10.1172/JCI121486 PMC6063472

[34] H. Chen , Z. Yang , C. Ding , L. Chu , Y. Zhang , K. Terry , H. Liu , Q. Shen , J. Zhou , Eur. J. Med. Chem. 2013, 62, 498. 23416191 10.1016/j.ejmech.2013.01.023 PMC3750725

[35] Q. Xie , Z. Yang , X. Huang , Z. Zhang , J. Li , J. Ju , H. Zhang , J. Ma , J. Hematol. Oncol. 2019, 12, 60. 31186039 10.1186/s13045-019-0744-3 PMC6558915

[36] D. E. Johnson , R. A. O'Keefe , J. R. Grandis , Nat. Rev. Clin. Oncol. 2018, 15, 234. 29405201 10.1038/nrclinonc.2018.8 PMC5858971

[37] H. Chen , Y. Chen , X. Wang , J. Yang , C. Huang , Free Radic. Res. 2020, 54, 351. 32543312 10.1080/10715762.2020.1772469

[38] L. Xiao , X. Li , P. Cao , W. Fei , H. Zhou , N. Tang , Y. Liu , J. Exp. Clin. Cancer Res. 2022, 41, 166. 35513871 10.1186/s13046-022-02376-4 PMC9069786

[39] D. Sun , W. Shen , F. Zhang , H. Fan , C. Xu , L. Li , J. Tan , Y. Miao , H. Zhang , Y. Yang , H. Cheng , Biomed. Pharmacother. 2018, 101, 107. 29477470 10.1016/j.biopha.2018.02.062

[40] O. Belmehdi , D. Taha , J. Abrini , L. C. Ming , A. Khalid , A. N. Abdalla , A. S. Algarni , A. Hermansyah , A. Bouyahya , Biomed. Pharmacother. 2023, 165, 115205. 37499451 10.1016/j.biopha.2023.115205

