# Supplementary material for: Discovery of Natural Compound α‐Hederin via Large‐Scale Screening as a Targeted JAK/STAT3 Inhibitor for Ovarian Cancer Therapy
Source: Adv Sci (Weinh). 2025 Jul 16;12(38):e17278. doi: 10.1002/advs.202417278 (PMC12520566; doi:10.1002/advs.202417278)
Supplement: Supplementary file 1 — Supporting Information [file ADVS-12-e17278-s001.docx]

**Supporting Information**

**Discovery of Natural Compound α-Hederin via Large-Scale Screening as a Targeted JAK/STAT3 Inhibitor for Ovarian Cancer Therapy**

Jiayu Wang^1#^, Pengzhan He^2#^, Cheng Liu^3#^, Xin Chen^3^, Yilin Tan^4^, Rui Qu^4^, Yan Zhang ^1*^, Zhou Li ^5*^, Tailang Yin^4*^, Zhinang Yin^1*^

**Supplementary figures**

**Fig. S1**

**
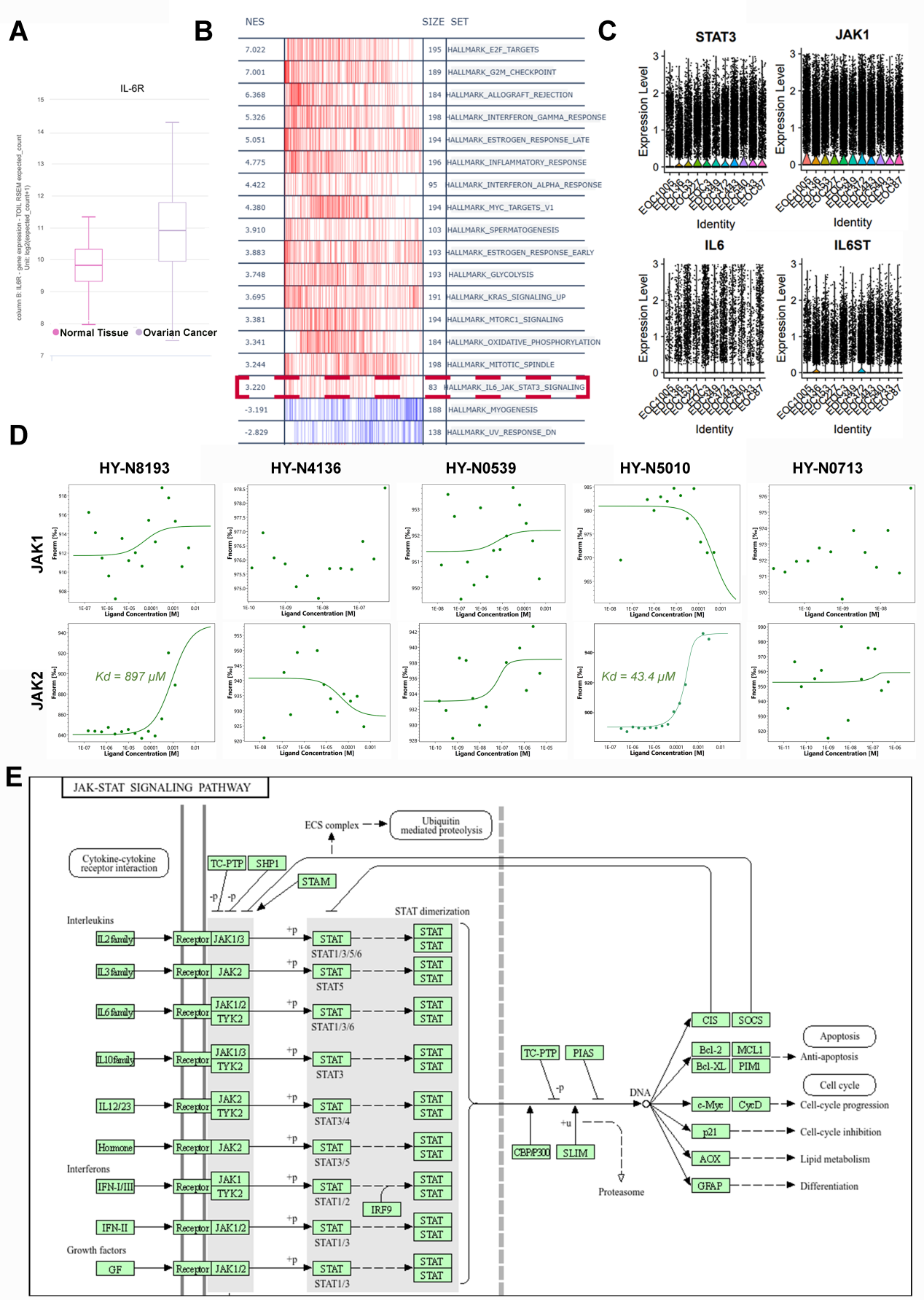
**

**Fig. S2
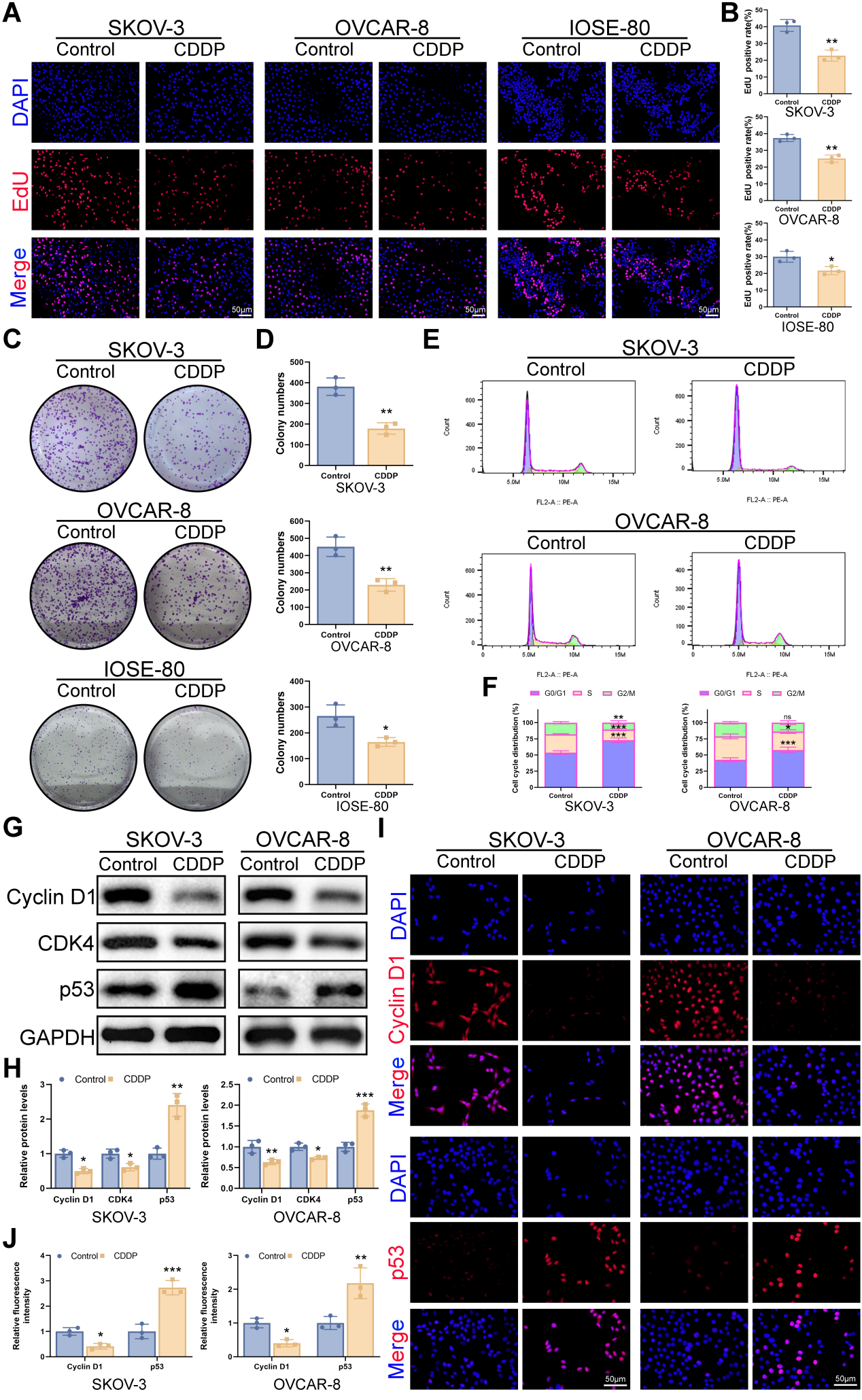
**

**Fig. S3**

**
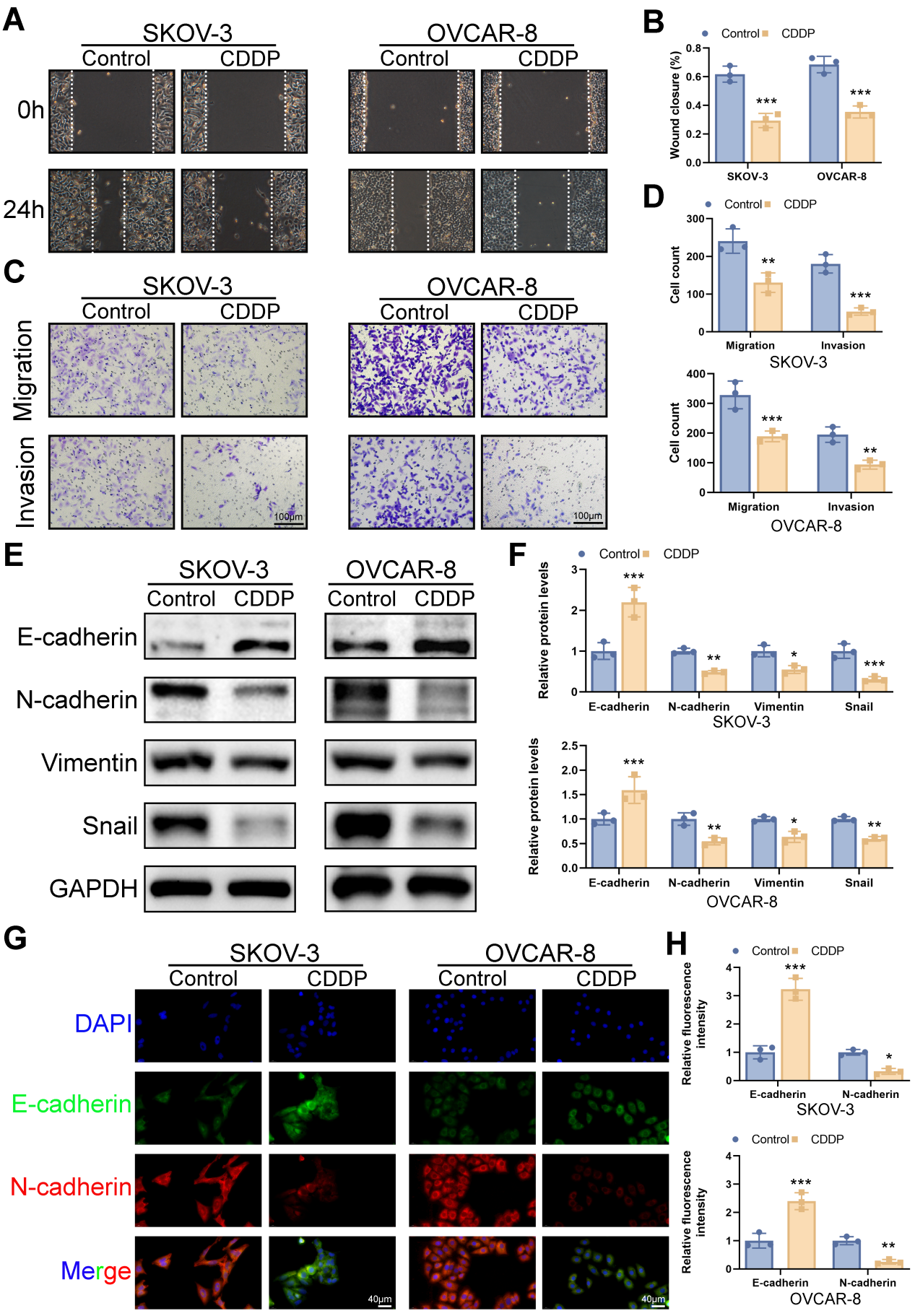
**

**Fig. S4**

**
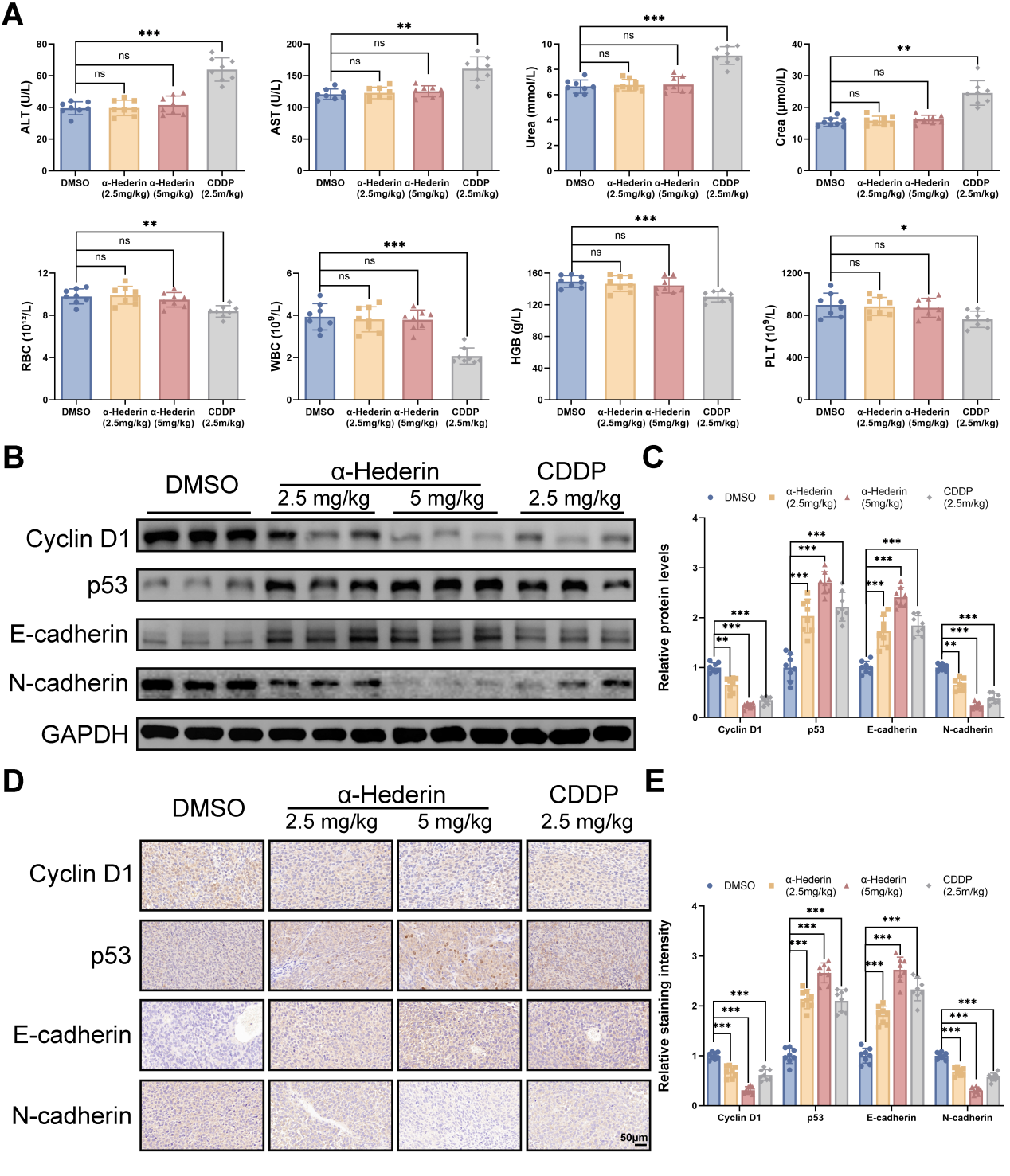
**

**Fig. S5**

**
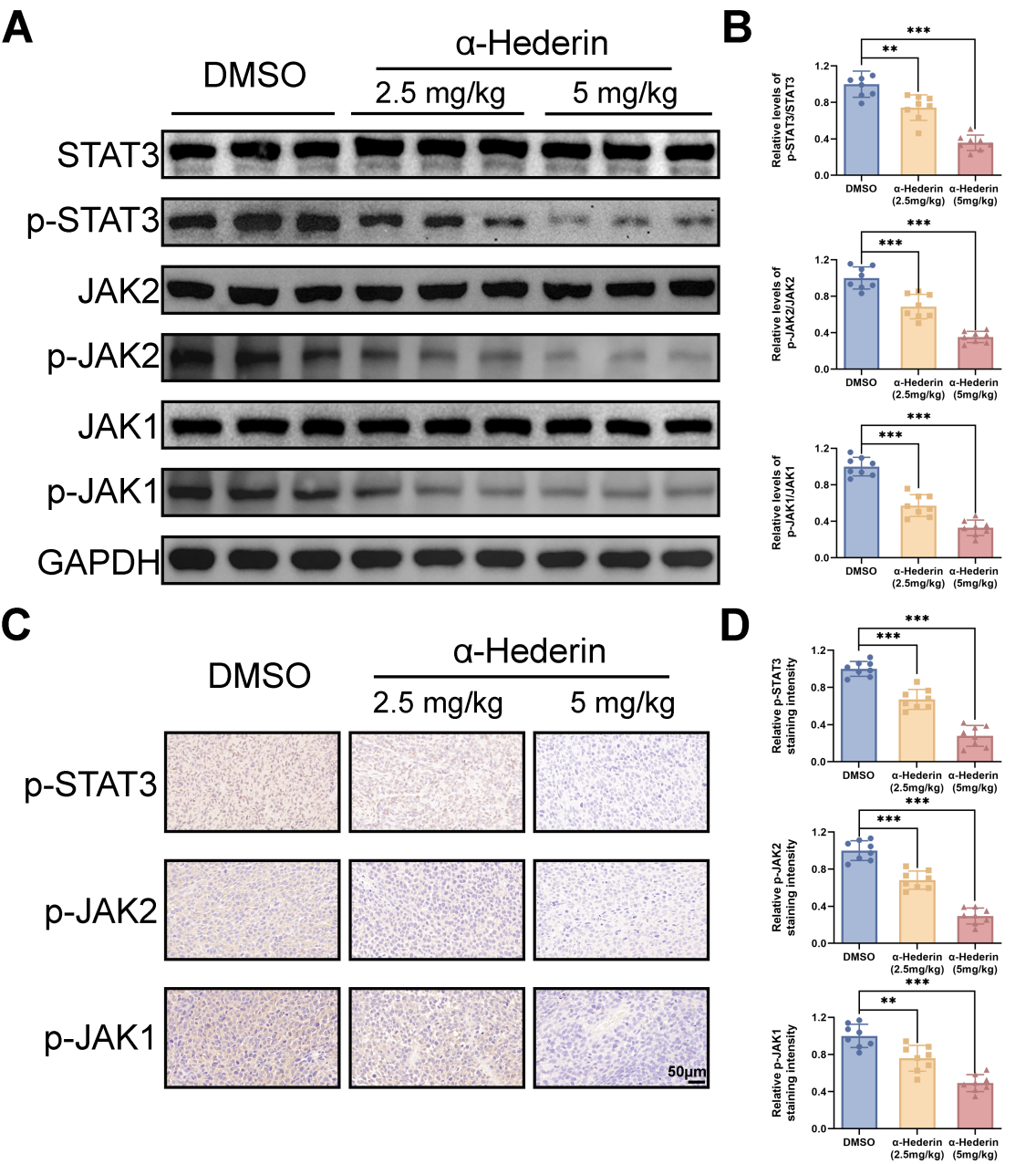
**

**Fig. S6**

**
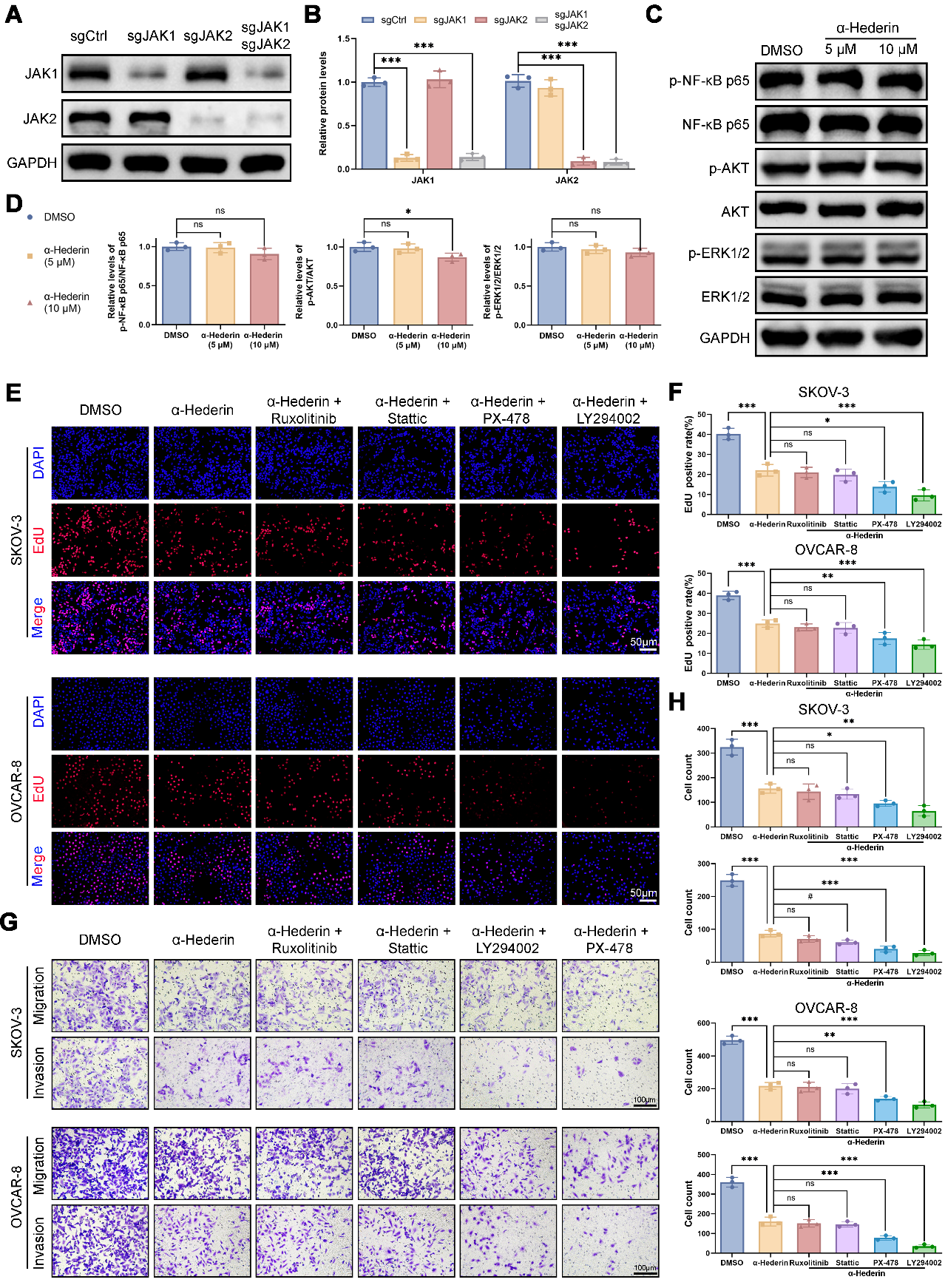
**

**Fig. S7**

**
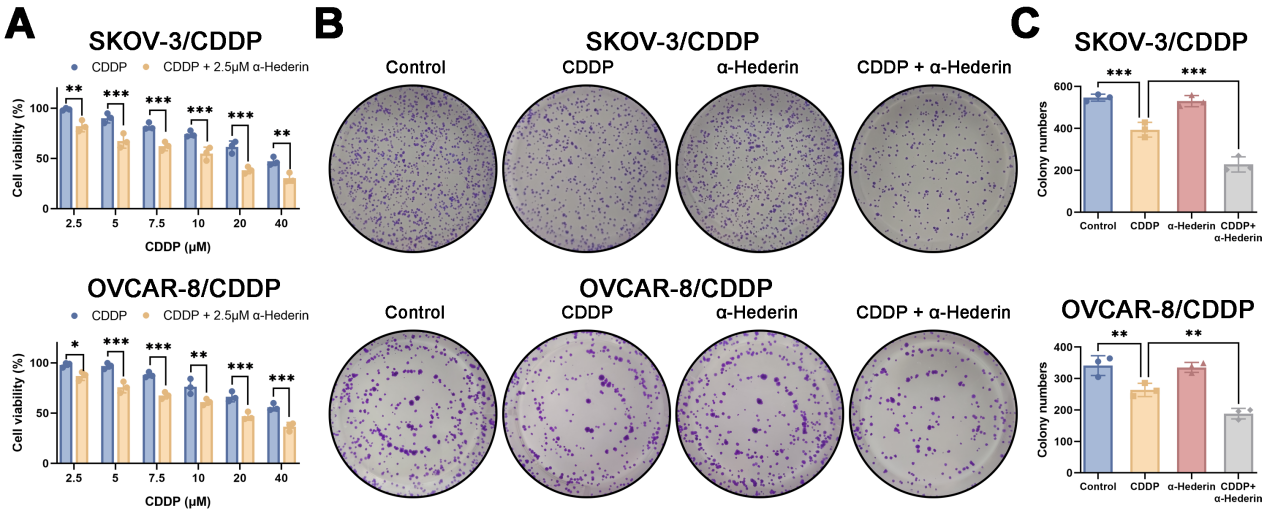
**

**Fig. S1 Bioinformatic analysis predicted that α-Hederin targets the JAK/STAT3 signaling pathway in OC.** (A) Boxplot showing increased mRNA expression of IL-6R in OC tissues compared to normal tissues, based on TCGA and GTEx datasets. . (B) GSEA list of pathways enriched in OC compared to normal ovary. The IL-6/JAK/STAT3 signaling pathway is highlighted within the red box, indicating its significant upregulation in OC. (C) Scatter plots showing the mRNA expression levels of IL6, IL6ST, STAT3, and JAK1 across individual OC patients. Each dot represents the expression level in a single cell, grouped by patient identity. (D) MST assay detecting the binding of selected natural compounds to JAK1 and JAK2. Shown are representative binding curves for each compound. (E) Schematic diagram of the JAK/STAT signaling pathway.

**Fig. S2 CDDP inhibits the proliferation of OC and IOSE-80 cells.** (A) Representative EdU staining images of SKOV-3, OVCAR-8, and IOSE-80 cells treated with 5 μM CDDP. Scale bar: 50 μm. (B) Quantification of EdU-positive cells from (A). (C) Representative images of colony formation assays in SKOV-3, OVCAR-8, and IOSE-80 cells following 5 μM CDDP treatment. (D) Quantification of colony numbers in (C). (E) Flow cytometry analysis of cell cycle distribution in SKOV-3 and OVCAR-8 cells treated with CDDP. (F) Quantification of cell cycle phase proportions (G0/G1, S, G2/M) from (E). (G-H) Protein levels of Cyclin D1, CDK4, and p53 were identified using Western blot, along with quantitative analysis. (I-J) IF staining of Cyclin D1, and p53 in SKOV-3 and OVCAR-8 cells, along with statistical analysis of fluorescence intensity. Scale bar: 50 μm. Data are presented as mean ± SD from three independent experiments. Statistical significance was evaluated using unpaired two-tailed Student’s t-test. Compared to Control: **p* < 0.05, ***p* < 0.01, ****p*< 0.001.

**Fig. S3** **CDDP significantly inhibits the migration and invasion of OC cells.** (A) Representative images from wound healing assays showing the migration of SKOV-3 and OVCAR-8 cells treated with CDDP (5 μM) for 24 h. (B) Quantification of wound closure percentage relative to initial gap width. (C) Transwell migration (top) and invasion (bottom) assays of CDDP-treated SKOV-3 and OVCAR-8 cells. Scale bar: 100 μm. (D) Quantification of migrated and invaded cells from (C). (E) Western blot analysis of EMT markers (E-cadherin, N-cadherin, Vimentin, and Snail) in cells treated with CDDP. (F) quantification of protein expression from (E). (G) IF staining of E-cadherin (green) and N-cadherin (red) in SKOV-3 and OVCAR-8 cells treated with CDDP for 24 h. Nuclei were stained with DAPI (blue). Scale bar: 40 μm. (H) Quantification of fluorescence intensity from (G). Data are presented as mean ± SD from three independent experiments. Statistical significance was assessed using unpaired two-tailed Student’s t-test. Compared to Control: **p* < 0.05, ***p* < 0.01, ****p*< 0.001.

**Fig. S4** **α-Hederin exhibits limited systemic toxicity and regulates cell cycle and EMT markers *in vivo*.** (A) Blood biochemical parameters and hematological indices of tumor-bearing mice treated with DMSO, α-Hederin (2.5 or 5 mg/kg), or CDDP (2.5 mg/kg) for 21 days. ALT, AST, urea, and creatinine were measured to assess liver and kidney function; RBC, WBC, HGB, and PLT counts were evaluated for hematopoietic toxicity (n = 8). (B) Western blot analysis of Cyclin D1, p53, E-cadherin, and N-cadherin expression in tumor tissues collected from treated mice. (C) Quantification of relative protein levels from (B). (D) Representative IHC staining of Cyclin D1, p53, E-cadherin, and N-cadherin in xenograft tumor sections. Scale bar: 50 μm. (E) Quantification of IHC staining intensity from (D). Data are presented as mean ± SD from eight mice per group (n = 8). Statistical significance was assessed using one-way ANOVA followed by multiple comparisons. **p* < 0.05, ***p* < 0.01, ****p*< 0.001, ns: not significant.

**Fig. S5** **α-Hederin suppresses phosphorylation of STAT3, JAK1, and JAK2 in xenograft tumors in a dose-dependent manner.** (A) Western blot analysis of total and phosphorylated STAT3, JAK1, and JAK2 in tumor tissues from mice treated with DMSO or α-Hederin (2.5 or 5 mg/kg). (B) Quantification of the relative phosphorylation levels of p-STAT3/STAT3, p-JAK2/JAK2, and p-JAK1/JAK1 from (A). (C) Representative IHC staining of p-STAT3, p-JAK2, and p-JAK1 in xenograft tumor tissues. Scale bar: 50 μm. (D) Quantitative analysis of IHC staining intensity from (C). Data are presented as mean ± SD from eight mice per group (n = 8). Statistical significance was assessed using one-way ANOVA followed by multiple comparisons. ***p* < 0.01, ****p*< 0.001.

**Fig. S6 α-Hederin inhibits STAT3 activation primarily via dual targeting of JAK1 and JAK2, with minimal effects on other oncogenic pathways.** (A) Western blot analysis confirming efficient CRISPR/Cas9-mediated knockout of JAK1 and/or JAK2 in SKOV-3 cells. (B) Quantification of JAK1 and JAK2 protein levels from (A). (C) Western blot showing expression levels of phosphorylated and total NF-κB p65, AKT, and ERK1/2 in SKOV-3 cells treated with α-Hederin (5 or 10 μM). (D) Quantification of p-NF-κB p65, p-AKT, and p-ERK1/2 levels from (C). (E) Representative EdU staining images of SKOV-3 and OVCAR-8 cells pretreated with selective inhibitors—Ruxolitinib, Stattic, LY294002, or PX-478—followed by α-Hederin treatment (10 μM) for 24 h. Scale bar: 50 μm. (F) Quantification of EdU-positive cells from (E). (G) Representative Transwell migration (top) and invasion (bottom) assays of SKOV-3 and OVCAR-8 cells pretreated as in (E), followed by α-Hederin treatment. Scale bar: 100 μm. (H) Quantitative analysis of migrated and invaded cell numbers from (G). Data are presented as mean ± SD from three independent experiments. Statistical significance was assessed using one-way ANOVA followed by multiple comparisons. **p* < 0.05, ***p* < 0.01, ****p*< 0.001, ns: not significant.

**Fig. S7** α**-Hederin restores CDDP sensitivity in CDDP-resistant OC cells.** (A) Cell viability of CDDP-resistant SKOV-3/CDDP and OVCAR-8/CDDP cells treated with CDDP alone or in combination with α-Hederin (2.5 μM). Cells were pretreated with α-Hederin for 2 h prior to CDDP exposure at the indicated concentrations for 48 h. (B) Representative images of colony formation assays in SKOV-3/CDDP and OVCAR-8/CDDP cells treated with DMSO, CDDP (10 μM), α-Hederin (2.5 μM), or the combination. (C) Quantification of colony numbers from (B). Data are presented as mean ± SD from three independent experiments. Statistical significance was evaluated using one-way ANOVA followed by multiple comparisons. **p* < 0.05, ***p* < 0.01, ****p*< 0.001.

**Supplementary tables**

**Table S1. Detailed information on the molecular docking of α-Hederin with JAK1**

| Lowest binding energy | | Binding energy  (Mean ± SD) | predicted Ki | Residue | AA |
| --- | --- | --- | --- | --- | --- |
|  |  | |  | 885A | HIS |
| -12.57  kcal/mol | -11.90± 0.38  kcal/mol | | 0.61  nM | 918A | HIS |
|  |  |  |  | 921A | ASP |
|  |  | |  | 1023A | GLY |

**Table S2. Detailed information on the molecular docking of α-Hederin with JAK2**

| Lowest binding energy | | Binding energy  (Mean ± SD) | predicted Ki | Residue | AA |
| --- | --- | --- | --- | --- | --- |
|  |  | |  | 867A | ARG |
| -12.10  kcal/mol | -10.76± 0.72  kcal/mol | | 1.34  nM | 877A | GLU |
|  |  |  |  | 914A | LYS |
|  |  | |  | 931A  985A | TYR  GLU |

**Table S3. The IC50 values of CDDP in the parental and CDDP-resistant OC cell lines**

| Cell lines | IC50 (μM) | RI |
| --- | --- | --- |
| SKOV-3 | 7.12 ± 0.37 | 4.64 |
| SKOV-3/CDDP | 33.08 ± 5.85 |  |
| OVCAR-8 | 10.95 ± 0.69 | 4.05 |
| OVCAR-8/CDDP | 44.37 ± 4.15 |  |

**RI: resistance index. Data are presented as mean ± SD.**
